# Self-control and performance while working from home

**DOI:** 10.1371/journal.pone.0282862

**Published:** 2023-04-13

**Authors:** Julia Baumann, Anastasia Danilov, Olga Stavrova

**Affiliations:** 1 School of Business and Economics, Humboldt-Universität zu Berlin, Berlin, Germany; 2 Department of Social Psychology, Tilburg University, Tilburg, Netherlands; Universitat Jaume I Departament d’Economia, SPAIN

## Abstract

This study explores the role of trait self-control in individuals’ changes in performance and well-being when working from home (WFH). In a three-wave longitudinal study with UK workers in the midst of the COVID-19 pandemic, we find that low self-control workers experienced a significant positive adjustment to WFH over time: The number of reported work distractions decreased, and self-assessed performance increased over the period of four months. In contrast, high self-control individuals did not show a similar upward trajectory. Despite the positive adjustment of low self-control individuals over time, on average, self-control was still positively associated with performance and negatively associated with work distractions. However, trait self-control was not consistently associated with changes in well-being. These findings provide a more nuanced view on trait self-control, suggesting that low self-control individuals can improve initial performance over time when working from home.

## Introduction

The rapid and forced transition to working from home (WFH) in 2020 represents one of the most disrupting and persistent changes that the COVID-19 pandemic brought about [1]. For example, 1.5 years into the pandemic (August 2021), half of the working population in the UK reported working from home at least on some of the days per week, which marks a 37% increase relative to the time before the pandemic [2]. How did this rapid and forced transition to WFH affect employees’ performance and well-being? This has been a central question in the research in social sciences since the beginning of the pandemic [3, 4]. Yet, research fell short of reaching a consensus, with studies portraying the transition to WFH as having either negative [5] or positive [4, 6] consequences for performance and well-being.

In the present research, we seek to reconcile these mixed findings by examining the role of individual differences in trait self-control–“the capacity to control impulses to resist a temptation […] and protect a valued goal” [7, p. 1117]–in the successful transition to WFH. A lack of clear structures, prevalence of distractions, temptations and procrastination opportunities are the hallmarks of WFH [8, 9]. The ability to deal with these distractions and exercise self-discipline is particularly important and is a common concern among teleworkers and their employers (see [10, 11] for anecdotal evidence from newspaper coverage). Hence, trait self-control might be particularly important for a successful working from home. Indeed, while some theoretical literature on the effectiveness of WFH in pre-pandemic times hinted at the importance of self-regulation [12], there is still a dearth of empirical work. In the present research, using three-wave longitudinal survey data of UK workers, we examined the role of trait self-control explaining temporal changes in performance (including performance quality and perceived work distractions) and well-being during the COVID-19 related transition to WFH.

### Related literature

Existing research exploring how the transition to WFH during the COVID-19 affected job outcomes has painted a mixed picture. Some studies revealed predominantly positive effects highlighting increased performance [6] and job satisfaction [13]. Other studies, on the contrary, found the transition to WFH to be associated with a deterioration in worker physical and mental health, presumably due to a lack of physical exercise during WFH [14], and decreasing performance, potentially driven by longer working hours with decreased focus [5]. A third group of studies detected no change in performance due to WFH policies [15].

Further studies have revealed a substantial degree of heterogeneity in how the transition to WFH affected different groups of workers. For example, in Etheridge et al. (2020) and Bellmann & Hübler (2020), WFH effects on performance and satisfaction varied depending on gender, income, and employment sector [13, 15]. Specifically, the performance of women and workers in the bottom income tier was lower when working from home compared to before the pandemic. Etheridge & Spantig (2022) found that women’s mental health in the early pandemic was particularly negatively affected compared to men [16]. This seemed to be driven largely by social factors, such as the stronger prevalence of extraversion in women, a personality trait associated with stronger declines in well-being during the pandemic.

Thus, the mixed findings regarding the average effect of WFH might be the result of heterogeneity in individuals’ adjustment to WFH, with some individuals showing more upward development in performance and well-being than others. Herein, we propose that individual differences in trait self-control could be important in explaining this between-individual heterogeneity.

Factors that matter for the success of WFH such as income, physical activity, a healthy diet, and work distractions (see [5, 12, 13]) have all been found to be positively correlated with trait self-control: Individuals scoring higher on trait self-control have been repeatedly shown to have better academic and work performance and better labor market outcomes including higher wages and lower unemployment rates [17–19]. High self-control individuals tend to exercise more and are more likely to follow a healthy diet compared to low self-control individuals [20, 21]. Finally, trait self-control has been associated with higher persistence at goal pursuit, lower susceptibility to temptations and more successful goal achievement [21, 22].

Self-control might be particularly important in the context of transition to WFH. Indeed, in a cross-sectional study of Chinese workers in early months of the pandemic, higher trait self-control was associated with less self-reported procrastination, less home-to-work interference, better performance, and higher life satisfaction [9]. Similarly, in a sample of German workers, Troll et al. (2021) showed that trait self-control predicted higher self-reported performance concurrently and over a period of one week [23]. Taken together, these previous findings suggest that individuals high in trait self-control would experience a better adjustment trajectory, showing increasing performance and well-being during the transition to WFH compared to individuals low in self-control.

The strong self-discipline associated with high self-control could also represent an obstacle, rather than an asset, in a successful adjustment during the transition to WFH. For example, prior research has often emphasized the importance of daily routines and stable structures as a way through which high self-control could lead to positive life outcomes [22, 24, 25]. Without these stable structures and routines, having high self-control may thus not be an advantage anymore. Applying this observation to the context of the COVID-19 pandemic, the transition to WFH brought about a disruption of daily routines and habits, suggesting that it could be associated with decreasing (rather than increasing) performance and well-being for high (vs. low) self-control individuals. Indeed, studies on self-control and flexibility of goal pursuit indicate that high self-control people do not show a higher flexibility and diversity in the use of self-control strategies than low self-control people [26]. In addition, there is also some evidence that low self-control people may have a better ability to disengage from unattainable goals, which could be beneficial at disrupting times of uncertainty and crisis (e.g., [27]). Finally, recent research on the role of conscientiousness–a personality trait closely linked to self-control (see e.g. [28])–suggests the possibility of detrimental effects of trait self-control during the WFH transition [29]. Specifically, Evans, Meyers, De Calseyde, and Stavrova (2021) showed that individuals high in conscientiousness reported deteriorating performance and well-being while working from home during the first 6 months of the pandemic [29]. Potentially, highly conscientious individuals could be particularly negatively affected by the lack of structures and disrupted daily routines during the transition to WFH. In line with this result, Bergefurt et al. (2022) found that conscientious employees became increasingly disengaged from their job in late 2020, potentially due to extended WFH [30]. Taken together, these previous findings suggest that individuals high in trait self-control might experience a worse adjustment trajectory, resulting in deteriorating performance and well-being during the transition to WFH than their low trait self-control counterparts.

## Materials and methods

In the present study, we examined the role of trait self-control in individuals’ adjustment to WFH during the COVID-19 pandemic. We took a longitudinal approach with three measurement points, documenting the effect of trait self-control at baseline on temporal changes in performance (self-rated performance quality and work distractions) and well-being (job satisfaction, life satisfaction and depression) over a four-month span in the midst of the pandemic in the spring and summer of 2021.

### Sample

We recruited participants via Prolific.co. Participants were residents of the UK who were full- or part-time employees and worked from home at least partially during the COVID-19 pandemic. The study included three measurement waves: The first wave was administered in March 2021, followed by the second wave one week later and the third wave in July 2021. Spring 2021 was a time when the pandemic had been causing restrictions to daily life for about a year (in Europe). The beginning of data collection in March 2021 was characterized by relatively strict virus containment measures in the UK, such as school closures and social distancing rules. Over the course of the study, these restrictions were gradually eased, culminating in the abandonment of all lockdown laws in mid-July [31].

Wave 1 data collection was followed by an intervention where participants were randomly assigned to follow one of four self-control strategies over the following week. However, the manipulation checks showed that the subjects did not follow the instructions; the manipulation had no effect on the manipulation check items or on any of the outcomes; even when controlling for experimental condition, they did not affect the results reported here. Therefore, we do not discuss the treatment effects further but refer the reader to the S1 File (S2) for details. The intervention study was conducted with an IRB approval (https://gfew.de/ethik/yNrddVjt) and registered in the AEA RCT Registry (AEARCTR-0007147). Participants consented to their participation in the study prior to filling out the survey. Wave 3 of data collection was not pre-registered. The results of the present study emerged from exploratory data analysis.

A gender-balanced sample of 200 women and 200 men (in total 400 participants) completed the first wave, whereas 262 participants completed all three waves. Participants were paid a total of £4.75 for the completion of all three surveys. Following the pre-registration, we excluded four subjects who did not pass the attention checks (see Section 3.2 for details) in at least one of the three surveys, resulting in the final sample of 258 participants. Participants who completed all three waves and scored higher on trait self-control (at baseline) were older and had more children, but did not differ with respect to other characteristics such as gender, compared to the participants who dropped out (see Table 1). 51% of participants in the final sample were female and participants’ average age was 36.69 years (*SD* = 10.12). Most participants reported holding a bachelor’s degree and earning a pre-tax income of £20,000 - £29,999; 30% had children. Before the pandemic, participants had spent an average of 15% (*SD* = 0.27) of their working hours working from home. At wave 1, they on average spent 88.91% (*SD* = 23.92) working from home. By wave 3, the share of time spent in the home office slightly decreased but remained relatively high: 72.49% (*SD* = 36.44).

**Table 1:** Summary statistics for the sample (N = 258) and check for differential attrition.

	Final sample N = 258		Dropout participants N = 138			
	*M*	*SD*	*M*	*SD*	*t / χ* ^*2*^	*p*
Gender	0.51	0.50	0.51	0.54	2.00	.37
Age	36.69	10.12	32.85	8.29	4.07	< .001
Children	0.30	0.46	0.00	0.00	49.24	< .001
Income	4.24	1.86	3.93	1.85	1.55	.12
Education	4.98	1.44	4.79	1.49	1.25	.21
Self-control t1	3.24	0.71	3.07	0.69	2.31	.02
% of time WFH before pandemic t1	15.16	27.30	16.96	29.47	-0.60	.55
% of time WFH during pandemic t1	88.91	23.92	87.88	23.52	0.42	.68
Contracted working hours t1	35.73	6.53	35.01	8.13	0.89	.37
Actual working hours t1	38.46	10.28	37.01	10.80	1.29	.20
Performance t1	4.45	0.52	4.43	0.55	0.30	.76
Work distractions t1	1.83	0.44	1.91	0.55	-1.49	.14
Life satisfaction t1	6.75	1.67	6.87	1.63	-0.70	.48
Job satisfaction t1	6.37	2.26	6.65	2.26	-1.18	.24
Depression t1	2.27	0.72	2.35	0.71	-1.04	.30

Gender: 1 = female, 0 = male; Children: 0 = no minor children living in the household, 1 = at least one minor child living in the household. Education, highest degree: 1 = Some high school, no diploma; 2 = High school graduate; 3 = Some college, no degree; 4 = Associate degree, 5 = Bachelor’s degree; 6 = Master’s degree; 7 = Professional degree; 8 = Doctorate degree. Income, pre-tax: 1 = Less than £10,000; 2 = £10,000 - £19,999; 3 = £20,000 - £29,999; 4 = £30,000 - £39,999; 5 = £40,000 - £49,999; 6 = £50,000 - £59,999; 7 = £60,000 - £69,999; 8 = £70,000 - £79,999; 9 = £80,000 - £89,999; 10 = £90,000 - £99,999; 11 = £100,000 - £149,999; 12 = More than £150,000; NA = Rather not say. 8 respondents marked “Rather not say” when asked about their income. t1 = wave 1. “t” indicates the test statistic from a two-tailed t-test or from a Chi-square test in the case of categorical variables (gender and children).

### Measures

#### Trait self-control

We measured self-control with the 13-item Tangney scale [28]. On a scale from 1 “Not at all” to 5 “Very much”, participants were asked to indicate their agreement with each item (e.g., “I am good at resisting temptation”, “I refuse things that are bad for me”). Items were reverse coded when necessary. Cronbach’s alpha was .88. The trait self-control was only measured at wave 1.

#### Performance

Performance was measured with the 7-item in-role behavior scale by Williams and Anderson (1991) [32]. Participants were asked to indicate how they currently felt about their performance in their job for each item on a 5-point Likert scale from 1 “Strongly disagree” to 5 “Strongly agree” (e.g., “I adequately complete assigned duties” and “I perform tasks that are expected of me”). This performance measure was included at all three waves. Cronbach’s alpha was .84 at wave 1, .81 at wave 2, and .83 at wave 3.

#### Work distractions

Given the importance of work distractions in WFH [30, 33], in addition to performance, we included a measure of perceived work distractions: “When working from home in the past seven days, how often have you experienced situations where you were distracted by the following?”. Participants were given a list of potentially distracting factors that they rated on a scale from 1 “Never” to 5 “Always”: Colleagues, child(ren), partner, housemate(s), pet(s), social media, instant messaging, news portals, noise, mind wandering, worries, household chores, the doorbell. We computed the average work distraction score across these different sources. The work distraction measure was administered in waves 1 and 3. Cronbach’s alpha reached .78 (wave 1) and .84 (wave 3).

#### Well-being

We used three different indicators of job-related and general psychological well-being. First, to measure job satisfaction, participants indicated their agreement with the item “How satisfied are you with your job?” on a scale from 0 “Extremely dissatisfied” to 10 “Extremely satisfied”. Second, we measured depression at all time points with the 8-item Centre for Epidemiological Studies Depression Scale (CES-D) [34]. For each item (e.g., “you felt depressed”, “you were happy” (reverse coded)), participants indicated how often they felt this way for the past seven days (1 “Never” to 5 “Always”). Cronbach’s alpha was .87 at wave 1, .88 at wave 2, and .89 at wave 3. Third, participants responded to a single-item measure of life satisfaction “How satisfied are you with your life, all things considered?” on a scale from 0 “Extremely dissatisfied” to 10 “Extremely satisfied”. All three measures of well-being were included in all three waves.

#### Control variables

To make sure that the effect of self-control is not due to a potential confounding with broader dimensions of personality, we additionally measured the Big Five personality traits. We used the Mini-IPIP [35] with a 5-point scale from 1 “Not at all” to 5 “Very much”, asking participants to indicate to what extent each statement reflected their typical behavior (e.g., “I am the life of the party”, “I have a vivid imagination”). Each Big Five trait was measured with four items. The Big Five scales were only included in wave 1. Cronbach’s alpha for each trait was .84 (Extraversion), .67 (Conscientiousness), .84 (Agreeableness), .77 (Neuroticism), .76 (Openness).

Participants also indicated what share of their contractual working hours they spent working from home (all time points). At wave 1, we also collected several socio-demographic variables: gender (1 = female, 0 = male), age, highest level of education (1 = Some high school, no diploma; 2 = High school graduate; 3 = Some college, no degree; 4 = Associate degree, 5 = Bachelor’s degree; 6 = Master’s degree; 7 = Professional degree; 8 = Doctorate degree), pre-tax income (1 = Less than £10,000; 2 = £10,000 - £19,999; 3 = £20,000 - £29,999; 4 = £30,000 - £39,999; 5 = £40,000 - £49,999; 6 = £50,000 - £59,999; 7 = £60,000 - £69,999; 8 = £70,000 - £79,999; 9 = £80,000 - £89,999; 10 = £90,000 - £99,999; 11 = £100,000 - £149,999; 12 = More than £150,000; 13 = Rather not say), and if there were any minor children living in the household (1 = yes, 0 = no).

### Empirical strategy

Since each participant contributed multiple data points (waves 1–3), we used a mixed effects approach where measurement waves are nested within participants. To examine whether baseline self-control predicts temporal changes in the outcome variables (performance, well-being, and work distractions), we estimated growth curve models with random intercepts at the level of participants. Time was measured as a continuous variable with values that reflect the number of weeks (since the start of the study) at each of the three waves (0, 1, 17.3).

We started by estimating a model with only the time variable as independent variable to examine the overall changes in performance and well-being over time (i.e., on average across the participants). Next, we proceeded to test the role of trait self-control as a potential moderator of the effect of time by examining the time × baseline self-control interactions. For each outcome (z-transformed), we estimated a model including the main effects of time and baseline self-control (z-transformed) as well as their interaction term. In a further model, we added socio-demographic variables (gender, age, children, income, education, and the share of time working from home) and Big Five personality traits (z-transformed) as control variables. The regression analyses were conducted using the *lme4* package in R [36]. Based on the regression results, we computed simple slopes of the effect of time at the mean level of self-control as well as one standard deviation below the mean and one standard deviation above the mean of self-control. This analysis follows the convention for the analysis of simple slopes established by Aiken and West (1991) [37]. Additionally, we computed the Johnson-Neyman intervals that show for which values of self-control the simple slopes are or would have been significant. To plot Johnson-Neyman intervals, the *interactions* package by Long (2019) [38] was used.

## Results

### Descriptive statistics

A full correlation matrix including (baseline) trait self-control and all outcome variables at all time points can be found in Table 2. In our sample, trait self-control was positively associated with performance, job satisfaction, and life satisfaction, and negatively associated with depression and work distractions at all time points.

**Table 2:** Means, standard deviations, and correlations with confidence intervals.

Variable	*M*	*SD*	1	2	3	4	5	6	7	8	9	10	11	12	13	14
1. Self-control t1	3.24	0.71														
2. Performance t1	4.45	0.52	.35***													
			[.23, .45]													
3. Performance t2	4.40	0.52	.32***	.53***												
			[.20, .42]	[.44, .61]												
4. Performance t3	4.51	0.51	.20**	.60***	.58***											
			[.08, .31]	[.52, .67]	[.49, .66]											
5. Job satisfaction t1	6.37	2.26	.22***	.34***	.23***	.15*										
			[.11, .34]	[.23, .44]	[.11, .34]	[.03, .27]										
6. Job satisfaction t2	6.60	2.07	.21***	.35***	.31***	.28***	.81***									
			[.10, .33]	[.24, .45]	[.20, .42]	[.16, .39]	[.77, .85]									
7. Job satisfaction t3	6.40	2.37	.25***	.30***	.16**	.25***	.66***	.68***								
			[.13, .36]	[.19, .41]	[.04, .28]	[.13, .36]	[.59, .73]	[.61, .74]								
8. Depression score t1	2.27	0.72	-.41***	-.28***	-.16**	-.18**	-.43***	-.41***	-.42***							
			[-.51, -.30]	[-.39, -.17]	[-.28, -.04]	[-.30, -.06]	[-.53, -.33]	[-.50, -.30]	[-.52, -.32]							
9. Depression score t2	2.16	0.69	-.39***	-.28***	-.23***	-.23***	-.38***	-.46***	-.47***	.85***						
			[-.49, -.28]	[-.39, -.16]	[-.34, -.11]	[-.34, -.11]	[-.48, -.27]	[-.55, -.36]	[-.56, -.37]	[.81, .88]						
10. Depression score t3	2.22	0.76	-.39***	-.26***	-.18**	-.25***	-.31***	-.35***	-.49***	.77***	.81***					
			[-.49, -.28]	[-.37, -.14]	[-.29, -.05]	[-.36, -.13]	[-.42, -.20]	[-.45, -.24]	[-.57, -.39]	[.71, .81]	[.76, .85]					
11. Life satisfaction t1	6.75	1.67	.30***	.26***	.10	.08	.52***	.50***	.54***	-.64***	-.67***	-.58***				
			[.18, .40]	[.15, .37]	[-.02, .22]	[-.04, .20]	[.43, .61]	[.40, .58]	[.45, .62]	[-.71, -.56]	[-.73, -.60]	[-.66, -.50]				
12. Life satisfaction t2	6.82	1.76	.27***	.18**	.14*	.13*	.38***	.46***	.45***	-.60***	-.65***	-.56***	.80***			
			[.15, .38]	[.06, .30]	[.02, .26]	[.01, .25]	[.27, .48]	[.36, .55]	[.34, .54]	[-.67, -.51]	[-.71, -.57]	[-.63, -.47]	[.75, .84]			
13. Life satisfaction t3	6.78	1.78	.35***	.23***	.10	.16*	.37***	.43***	.58***	-.60***	-.64***	-.69***	.77***	.73***		
			[.23, .45]	[.11, .34]	[-.03, .21]	[.03, .27]	[.26, .47]	[.33, .53]	[.49, .65]	[-.68, -.52]	[-.71, -.57]	[-.75, -.62]	[.72, .82]	[.67, .78]		
14. Work distractions t1	1.83	0.44	-.37***	-.35***	-.31***	-.29***	-.12*	-.11	-.16**	.41***	.37***	.38***	-.14*	-.10	-.17**	
			[-.47, -.26]	[-.46, -.24]	[-.41, -.19]	[-.40, -.18]	[-.24, -.00]	[-.23, .01]	[-.28, -.04]	[.30, .51]	[.26, .47]	[.27, .48]	[-.26, -.02]	[-.22, .02]	[-.28, -.05]	
15. Work distractions t3	1.75	0.46	-.25***	-.33***	-.35***	-.36***	-.07	-.12*	-.10	.29***	.28***	.31***	-.11	-.04	-.10	.70***
			[-.36, -.13]	[-.43, -.21]	[-.46, -.24]	[-.46, -.24]	[-.19, .05]	[-.24, -.00]	[-.22, .02]	[.17, .40]	[.16, .39]	[.19, .42]	[-.23, .01]	[-.16, .09]	[-.22, .02]	[.63, .75]

*M* and *SD* represent mean and standard deviation, respectively. Values in square brackets indicate the 95% confidence interval for each correlation. * indicates *p* < .05. ** indicates *p* < .01, *** indicates *p* < 0.001.

### Temporal trajectories of performance and well-being

**Fig 1:**
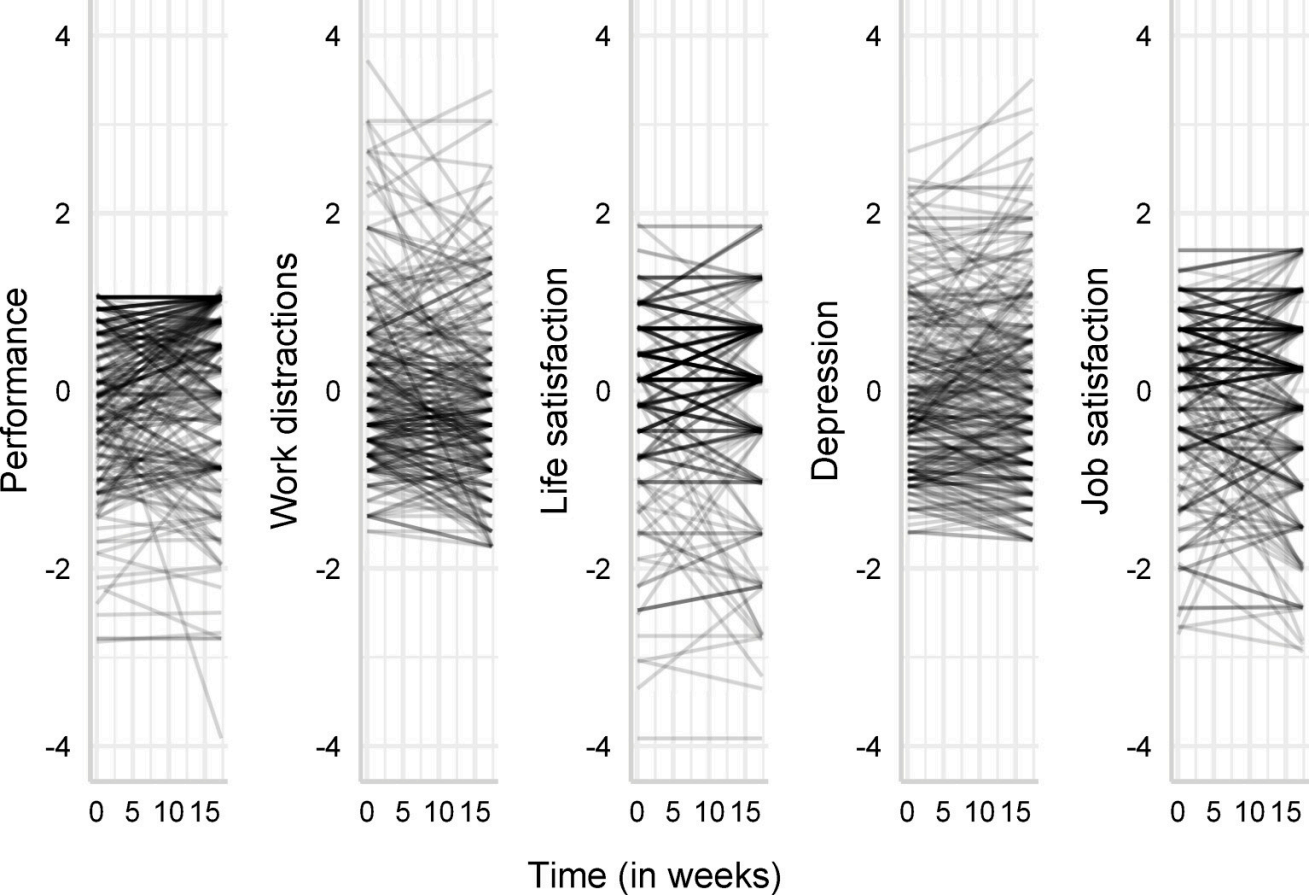
Between-individual heterogeneity in temporal development in worker outcomes. Each line represents a slope of time computed for each individual. Outcomes are z-transformed.

First, we estimated the average temporal trajectory of the outcome variables by regressing each outcome on the time variable (Table 3). The models included random intercepts for each outcome variable. On average, participants’ performance increased over time (*b* = 0.010, 95% CI [0.004,0.015], *p* = .001), while work distractions decreased (*b* = −0.011, 95% CI [−0.016, −0.005], *p* < .001). The total change amounts to an increase in performance of 0.173 standard deviations and a total decrease in work distractions of 0.1903 standard deviations between wave 1 and wave 3 (i.e., over four months). We did not observe significant changes over time in life satisfaction, depression and job satisfaction (life satisfaction: *b* = 0.000, 95% CI [−0.004,0.004], *p* = .964; depression: *b* = 0.000, 95% CI [−0.004,0.004], *p* = .904; job satisfaction: *b* = −0.002, 95% CI [−0.007,0.003], *p* = .509). Fig 1 shows that there was a substantial between-individual difference in the temporal trajectories of the outcome variables. In the next section, we explore whether part of this heterogeneity can be explained by differences in trait self-control at baseline (t1).

**Table 3:** Effect of time on worker outcomes.

	Performance	Work distractions	Life satisfaction	Depression	Job satisfaction
	*b* 95% CI [LL,UL]	*b* 95% CI [LL,UL]	*b* 95% CI [LL,UL]	*b* 95% CI [LL,UL]	*b* 95% CI [LL,UL]
Intercept	-0.059 [-0.168, 0.051]	0.091 [-0.031, 0.213]	-0.001 [-0.117, 0.115]	-0.002[-0.116, 0.113]	0.012 [-0.102, 0.126]
Time	0.010** [0.004, 0.015]	-0.011*** [-0.016, -0.005]	0.000 [-0.005, 0.005]	0.000 [-0.004, 0.005]	-0.002 [-0.008, 0.004]
AIC	1978.4	1310.9	1706.4	1623.2	1768.7
BIC	2006.3	1327.9	1734.3	1651.1	1796.6
Log.Lik.	-983.209	-651.455	-847.210	-805.581	-878.333
REMLcrit	1966.418	1302.910	1694.420	1611.162	1756.665

Results obtained from a linear mixed effects model with individual intercepts and slopes. 95% confidence intervals are in brackets. All outcome variables are z-transformed. * *p <* .*05*, ^**^ *p <* .*01*, ^***^ *p*<0.001.

### Baseline self-control and between-individual heterogeneity in outcome development over time

To test whether individual differences in trait self-control at baseline can explain different trends in the outcome variables over time, we estimated a series of models including the measure of baseline self-control, time and the interaction time × baseline self-control. The results are presented in Table 4.

**Table 4:** Effect of time on worker outcomes as a function of baseline self-control.

	Performance		Work distractions		Life satisfaction	
	Model 1	Model 2	Model 1	Model 2	Model 1	Model 2
	*b* 95% CI [LL,UL]	*b* 95% CI [LL,UL]	*b* 95% CI [LL,UL]	*b* 95% CI [LL,UL]	*b* 95% CI [LL,UL]	*b* 95% CI [LL,UL]
Intercept	-0.059 [-0.160, 0.043]	0.069 [-0.477, 0.614]	0.091 [-0.025, 0.207]	0.518 [-0.061, 1.097]	-0.001 [-0.111, 0.110]	-0.223 [-0.795, 0.350]
Time	0.010** [0.004, 0.015]	0.011*** [0.004, 0.017]	-0.011*** [-0.016, -0.005]	-0.009** [-0.015, -0.003]	0.000 [-0.005, 0.005]	-0.001 [-0.006, 0.004]
Self-control	0.335*** [0.234, 0.436]	0.272*** [0.153, 0.391]	-0.361*** [-0.477, -0.244]	-0.312*** [-0.443, -0.181]	0.274*** [0.164, 0.385]	0.071 [-0.053, 0.195]
Time × self-control	-0.008** [-0.014, -0.002]	-0.008* [-0.013, -0.002]	0.006* [0.000, 0.011]	0.007* [0.001, 0.012]	0.005 [0.000, 0.009]	0.004 [0.000, 0.009]
Gender		0.273* [0.058, 0.489]		-0.118 [-0.347, 0.111]		0.284* [0.057, 0.510]
Age		-0.004 [-0.013, 0.006]		-0.021*** [-0.031, -0.011]		-0.002 [-0.012, 0.008]
Children		-0.159 [-0.370, 0.052]		0.250* [0.026, 0.474]		0.186 [-0.036, 0.407]
Income		0.020 [-0.033, 0.074]		0.014 [-0.043, 0.071]		0.033 [-0.024, 0.090]
Education		-0.030 [-0.098, 0.038]		0.048 [-0.024, 0.121]		-0.015 [-0.087, 0.056]
% working from home		-0.006 [-0.079, 0.066]		0.048 [-0.031, 0.127]		-0.022 [-0.084, 0.039]
Extraversion		0.014 [-0.093, 0.121]		0.143* [0.029, 0.256]		0.105 [-0.008, 0.217]
Agreeableness		-0.007 [-0.111, 0.098]		0.074 [-0.037, 0.185]		0.034 [-0.076, 0.144]
Conscientiousness		0.107 [-0.001, 0.216]		0.018 [-0.097, 0.133]		0.087 [-0.026, 0.201]
Neuroticism		-0.036 [-0.145, 0.074]		0.178** [0.062, 0.295]		-0.382*** [-0.498, -0.267]
Openness		0.064 [-0.036, 0.164]		-0.013 [-0.119, 0.092]		-0.077 [-0.182, 0.028]
AIC	1956.2	1928.2	1293.3	1268.0	1691.7	1657.3
BIC	1993.4	2016.0	1318.8	1339.6	1728.9	1745.1
Log.Lik.	-970.108	-945.088	-640.650	-616.998	-837.843	-809.658
REMLcrit	1940.216	1890.175	1281.300	1233.996	1675.685	1619.316

Results obtained from a linear mixed effects model with individual intercepts and slopes. 95% confidence intervals are in brackets. Outcome variables, self-control and Big Five personality traits are z-transformed.

*p < .05

**p < .01

***p<0.001. In Model 2, the number of observations in the regression is slightly lower due to missing values in the income variable, dropping from 258 individuals in Model 1 to 250 individuals.

#### Performance

**Fig 2:**
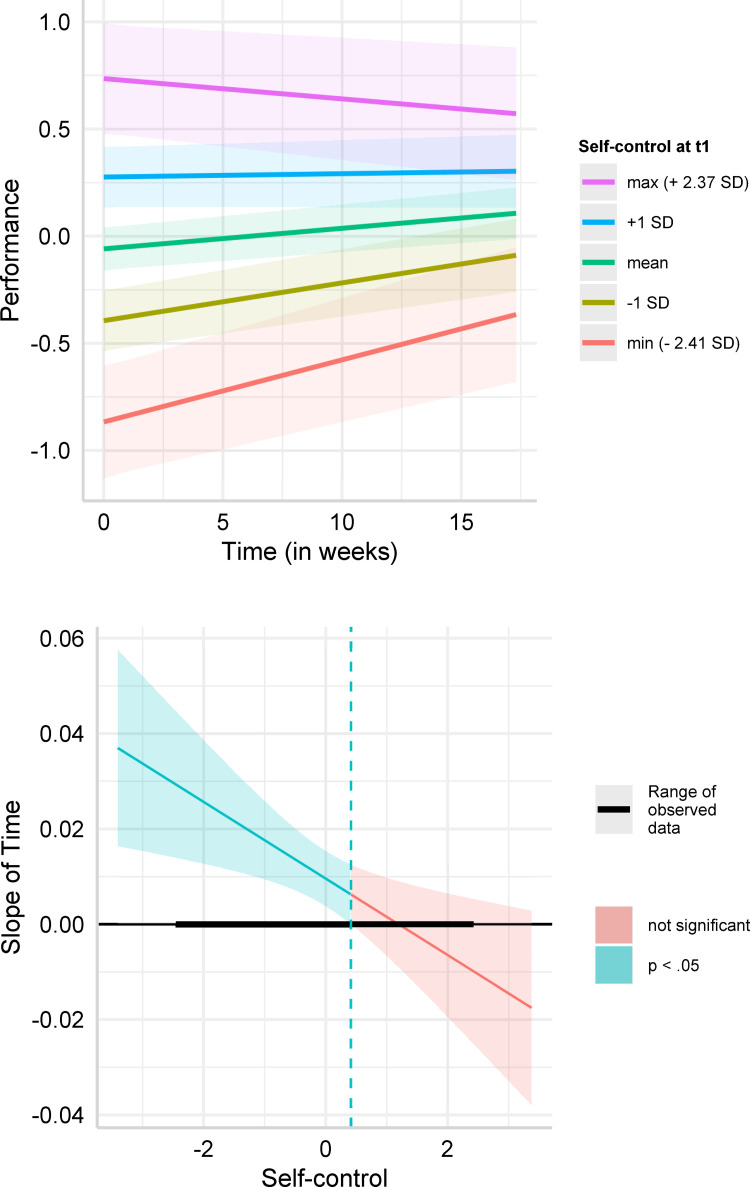
**A.** Predicted values of performance. Simple slope estimates for Model 1 (dependent variable: performance) in Table 4. The figure depicts predicted values of performance (standardized around 0) over time for low self-control (-1 SD below mean), average self-control, high self-control (+1 SD above mean) individuals, minimum self-control in the sample (-2.41 SD), and maximum self-control in the sample (2.37 SD). **B.** Johnson-Neyman intervals for performance. Johnson-Neyman intervals for the simple slope estimates for Model 1 (dependent variable: performance) in Table 4. The figure depicts the estimated slope of time for each level of trait self-control (standardized around 0) and highlights for which values of trait self-control the simple slope estimate is significant.

We detected a significant negative interaction effect between time and self-control (*b* = −0.008, 95% CI [−0.014, −0.002], *p* = .007). This effect remained robust when adding all the control variables in Model 2 (*b* = −0.008, 95% CI [−0.013, −0.002], *p* = .013). Fig 2A plots the change in performance over time for individuals with low (one standard deviation below mean) and high (one standard deviation above mean) baseline level of trait self-control, as well as at the mean (0 SD), the minimum (-2.41 SD) and the maximum trait self-control (2.37 SD) in the sample, based on Model 1. In the S1 File, we also report the simple slopes for self-control +2 SD and -2 SD around the mean (S16 Table, S1-S3 Figs in S1 File). These slopes show that low self-control individuals experienced an increase in performance over time (-1 SD: *b* = 0.018, *p* < .001; minimum: *b* = 0.029, *p* < .001), whereas the performance of high self-control individuals did not significantly change (+1 SD: *b* = 0.002, *p* = .710; maximum: *b* = −0.009, *p* = .214). This implies that individuals with self-control one standard deviation below the mean experienced on average a significant increase of 0.311 (= 0.018*17.3 [weeks]) standard deviations in performance over the course of the study. In contrast, individuals with self-control one deviation above the mean did not experience any significant change in performance over the same period. We additionally computed the Johnson-Neyman significance region, i.e., a range of self-control values within which we observed significant temporal changes in performance (Fig 2B). A total of 163 individuals whose trait self-control ranged between the lowest possible score and 0.41 standard deviations above the mean experienced an increase in performance over time, while 95 individuals whose self-control was above that value did not experience any change in performance. An examination of the effect of self-control on performance at three waves separately showed that performance of low self-control individuals converged towards the performance of high self-control individuals over time, while still remaining significantly lower (wave 1: *b* = 0.345, 95% CI [0.230,0461], *p* < .001, wave 2: *b* = 0.316, 95% CI [0.199,0.432], *p* < .001, wave 3: *b* = 0.199, 95% CI [0.079,0.320], *p* = .001; see S1 File (S3 Table) for further details).

Even though the performance scale used in this study is a validated measure for in-role productivity [32], potential ceiling effects may be problematic for high self-control individuals who already start out with a high performance in wave 1 and may be the reason that high self-control individuals did not show any adjustment in our study. We conducted several additional analyses to address this possibility. First, though a part of the sample is at the upper bound of the performance measure (= 5), even for high self-control individuals (+1 SD above mean and more), the average performance is still significantly different from the upper bound (*M* = 4.71, *p* < 0.001). Second, we replicated our results using a Tobit model for censored data which has been shown to provide unbiased estimated in the presence of ceiling effects [39]. Consistent with the main results, the interaction between time and self-control was significant (b = - 0.01, 95% CI [-0.017,-0.002], p = .014). The Johnson-Neyman intervals are likewise only slightly affected, indicating that the simple slopes are significant for self-control levels 0.48 standard deviations above the mean (main specification: 0.41 standard deviations above the mean). Detailed results can be found in the S1 File (S17 Table and S4 Fig).

#### Work distractions

**Fig 3:**
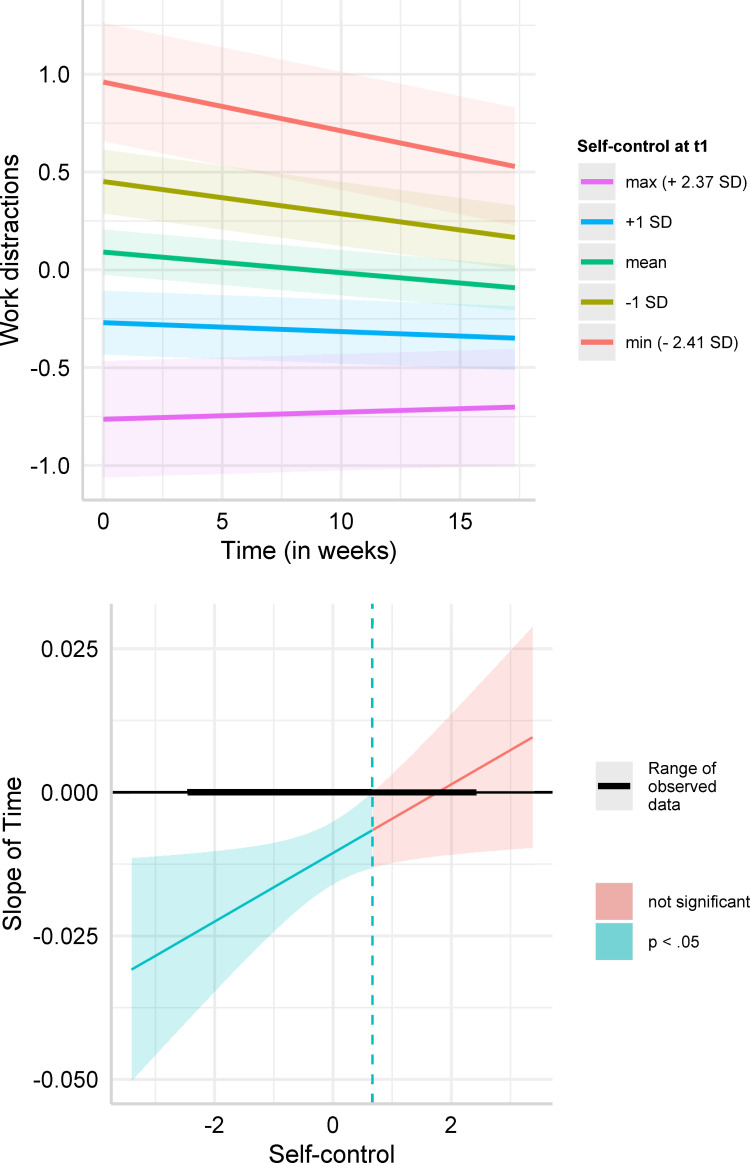
**A.** Predicted values of work distractions. Simple slope estimates for Model 1 (dependent variable: work distractions) in Table 4. The figure depicts predicted values of work distractions (standardized around 0) over time for low self-control (-1 SD below mean), average self-control, high self-control (+1 SD above mean) individuals, minimum self-control in the sample (-2.41 SD), and maximum self-control in the sample (2.37 SD). **B.** Johnson-Neyman intervals for work distractions. Johnson-Neyman intervals for the simple slope estimates for Model 1 (dependent variable: work distractions) in Table 4. The figure depicts the estimated slope of time for each level of trait self-control (standardized around 0) and highlights for which values of trait self-control the simple slope estimate is significant.

We also detected a significant interaction effect between time and self-control on work distractions (*b* = 0.006, 95% CI [0.000,0.011], *p* = .033). The coefficient implies a significant decrease in work distractions of 0.104 (= 0.006*17.3 [weeks]) standard deviations for individuals with trait self-control one standard deviation below the mean. In contrast, individuals with high self-control did not experience any change in work distractions. This effect was robust to adding control variables to the regression model (Model 2; *b* = 0.007, 95% CI [0.001,0.012], *p* = .022). Fig 3A plots the change in work distraction scores over time for low (one standard deviation below mean and minimum), high (one standard deviation above mean and maximum) and mean self-control individuals, based on Model 1. It demonstrates that the work distraction score decreased over time for low self-control individuals (-1 SD: *b* = −0.017, *p* < .001; minimum: *b* = −0.025, *p* = .001) while it did not change significantly for high self-control individuals (+1 SD: *b* = −0.005, *p* = .247; maximum: *b* = 0.004, *p* = .615). The Johnson-Neyman intervals (Fig 3B) show that individuals with a self-control score below 0.67 standard deviations (N = 200) above the mean experienced a decrease in work distractions over time, while individuals with trait self-control score higher than that (N = 58) did not experience any change. Examining the effect of self-control on work distractions at wave 1 and wave 3 separately, showed that the effect of self-control was lower at wave 3. Thus, work distractions of high and low self-control individuals converged over time, while still remaining significantly higher overall for low self-control individuals (wave 1: *b* = −0.371, 95% CI [−0.485, −0.256], *p* < .001, wave 3: *b* = −0.252, 95% CI [−0.372, −0.133], *p* < .001; see S1 File (S1 Table) for further details).

#### Well-being

**Fig 4:**
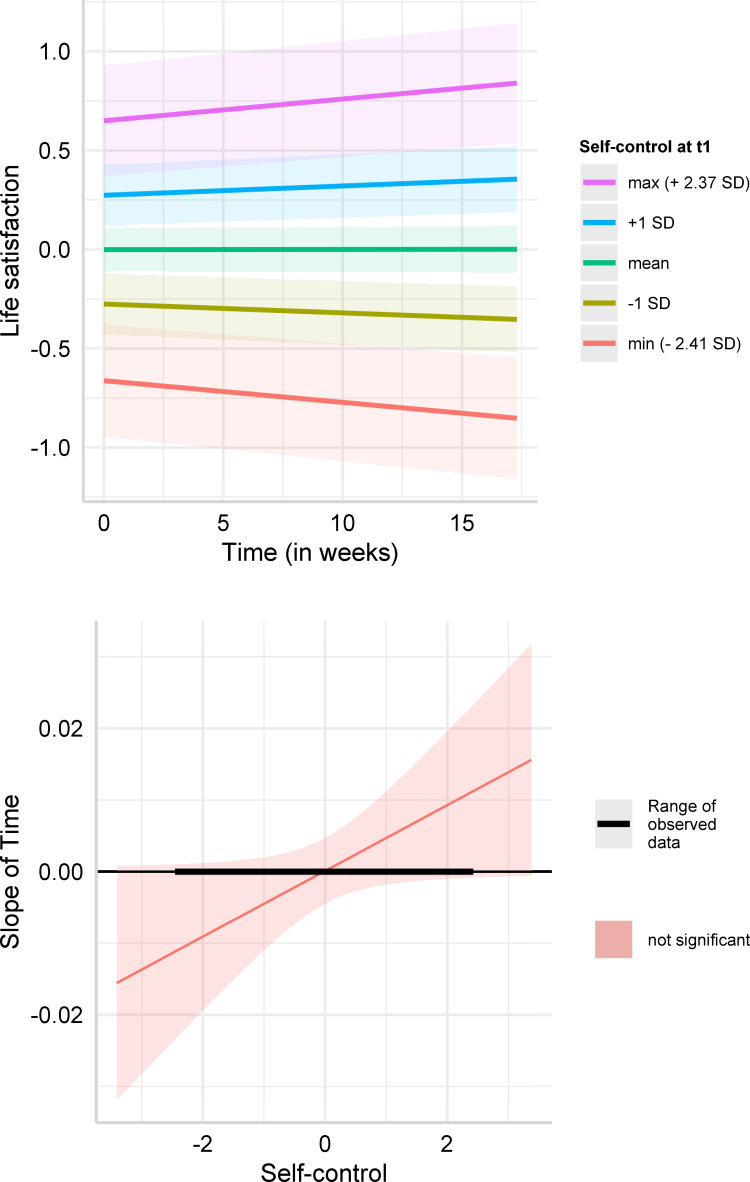
**A.** Predicted values of life satisfaction. Simple slope estimates for Model 1 (dependent variable: life satisfaction) in Table 4. The figure depicts predicted values of life satisfaction (standardized around 0) over time for low self-control (-1 SD below mean), average self-control, high self-control (+1 SD above mean) individuals, minimum self-control in the sample (-2.41 SD), and maximum self-control in the sample (2.37 SD). **B.** Johnson-Neyman intervals for life satisfaction. Johnson-Neyman intervals for the simple slope estimates for Model 1 (dependent variable: life satisfaction) in Table 4. The figure depicts the estimated slope of time for each level of trait self-control (standardized around 0) and highlights for which values of trait self-control the simple slope estimate is significant.

We did not find a significant interaction effect between time and self-control on life satisfaction (*b* = 0.005, 95% CI [0.000,0.009], *p* = .051; Model 2: *b* = 0.004, 95% CI [0.000,0.009], *p* = .079). Fig 4A plots the change in life satisfaction over time for low (one standard deviation below mean) and high (one standard deviation above mean) levels of self-control, based on Model 1. Life satisfaction had a tendency to increase for high self-control individuals and decrease for low self-control individuals, though the simple slopes did not reach significance at any self-control level (minimum: *b* = −0.011, *p* = 0.055; -1 SD: *b* = −0.004, *p* = 0.177; +1 SD: *b* = 0.005, *p* = 0.157; maximum: *b* = 0.011, *p* = 0.051). The Johnson-Neyman intervals (Fig 4B) showed that there was no significant simple slope within the observed range of self-control. The interaction effect did not replicate for the other well-being outcomes either (depression and job satisfaction; we report these regressions in the S1 File (S2 Table)).

#### Control variables

Regarding the control variables in Model 2, we note that none of them could explain the positive adjustment of low self-control individuals. We controlled for Big Five personality traits to avoid potential confounding factors since these personality traits may impact our outcome variables of interest. Big Five personality traits were correlated with self-control as follows: .15* [.03, .27] (Extraversion), .17** [0.05, 0.29] (Agreeableness), .48** [.38, .57] (Conscientiousness), -.36** [-.47, -.25] (Neuroticism), -.05 [-.17, .07] (Openness). Despite these correlations, controlling for personality traits did not affect the interaction effect Self-control x time. Further, as the number of hours worked from home vs. office changed over time, we tested whether the share of work time spent at home was correlated with the reported work-related distractions. In our data, we find no significant correlation between these two variables (ρ = 0.058, p = 0.192). By controlling for the share of WFH time in the main regressions, we rule out the possibility that the positive adjustment experienced by low self-control individuals is explained by them going back to the office more frequently where they might face different working conditions than at home. We also note that controlling for having children did not change the main results in any way, ruling out the possibility of the positive adjustment of low self-control individuals being driven by improved access to childcare over the course of the study period.

## Discussion

The massive transition to WFH represented one of the major changes in work life for the years 2020–2021. While self-control–a personality dimension central to individuals’ ability to deal with work distractions and exercise self-discipline–is often discussed as an important ingredient of successful WFH outcomes [9, 23], there is still a lack of empirical research on the effect of trait self-control on the adjustment to WFH. In the present study, we attempted to fill this research gap by adopting a longitudinal research design, following UK workers over a four-month period in the midst of the pandemic. We found that trait self-control is generally positively associated with performance and well-being, and negatively associated with work distractions during WFH. Over time, however, we observed a convergence in the performance of low self-control and high self-control individuals: low self-control workers experienced a significant improvement in performance (including reporting fewer work distractions) over the course of 17 weeks, compared to high self-control individuals. This improved performance is evidence that low self-control individuals where able to adjust to WFH. While performance increased over time for low self-control individuals, well-being did not exhibit the same trajectory. Potentially, experiencing less work distractions was associated with more job stress for low self-control workers, preventing them from experiencing rising well-being during the WFH transition. This possibility is consistent with previous research that showed that decreasing work distractions improved productivity but increased perceived stress at work [40].

### Theoretical implications

Extant literature has painted an overwhelmingly negative picture of low self-control individuals in the workplace, highlighting their lower performance, less successful goal achievement, and slower career advancement [17–19, 21, 22]. In contrast, the present study is among the first to provide a more calibrated, optimistic view of low self-control: We showed that–during the pandemic-induced transition to WFH–low self-control workers experienced a convergence toward the performance of high self-control workers, as evidenced by decreasing work distractions and rising performance. Even though high self-control is beneficial in regular times, low self-control might offer advantages during times of transition, uncertainty, and crisis [41]. Potentially, low self-control individuals may have an easier time adjusting to the new circumstances, for example, by setting more realistic goals. Indeed, prior research attributes more goal flexibility to low (vs. high) self-control individuals [27], showing that low self-control is associated with a lower tendency to set unattainable goals, improving performance [42]. One further reason we didn’t detect any change among high self-control individuals may be diminishing marginal productivity due to a very high-performance level to begin with. Arguably, high self-control individuals’ performance remained high throughout the study period. Taken together, the present results contribute to our understanding of the relationship between trait self-control and job outcomes, adding to the emergent literature on the potential downsides of high and upsides of low self-control [43].

### Limitations and future research directions

The results of the present study emerged from exploratory data analysis; this data set had originally been collected for other purposes (see Section Materials and Methods and S1 File). Furthermore, when considering the results of this study in a larger context, it is important to note that even though the data collected covered a considerably long time period during the pandemic (four months), it remains to be explored whether the upward performance trajectory experienced by low self-control workers would extend to post-pandemic times, further contributing to closing the performance gap between high and low self-control individuals. On a related note, future studies should examine whether the positive effect of low self-control is restricted to the pandemic context versus can be observed during transitions to WFH more generally.

Since our data collection started when WFH policies were already in place, we have no information about how performance and other outcomes during the study compare to pre-WFH levels. However, we did collect data on the share of time participants worked from home before the COVID-19 pandemic. If the adjustment effect among low self-control workers was due to them being particularly burdened by the transition to remote work at the beginning of the pandemic, we would expect to find improved performance mostly among workers who were new to the WFH arrangement. To test this possibility, we checked whether the interaction between self-control and time was further qualified by the share of time participants worked from home before the pandemic (“What share of your working hours did you use to work from home before the Covid-19 pandemic?”, 0–100). The three-way interaction was not significant (*p* = 0.811), suggesting that the adjustment pattern did not depend on whether the workers had prior working from home experience or not. These additional results are shown in the S1 File (S18 Table). This analysis provides some initial indication that the adjustment effect found in this study is unlikely to represent a recovery experienced by low self-control individuals after the initial shock.

A potential technical limitation of our results may be a ceiling effect in the performance measure. As discussed in the Results section, the average performance score is close to the upper bound of 5. Even though the performance scale used in this study is a validated measure for in-role productivity [32], this is potentially problematic for high self-control individuals who already start out with a high performance in wave 1 and may be the reason that high self-control individuals did not show any adjustment in our study. We therefore conducted additional analyses, including estimating a Tobit model for censored regressions [39]. Our results remained robust to this analysis. We therefore take this as suggestive evidence that high self-control individuals actually did not adjust to WFH conditions, while low self-control individuals did.

Finally, while the present results provide first evidence of the beneficial effect of low self-control on the adjustment to WFH, our data fell short of unravelling the exact mechanism of this effect. Our results did not seem to be driven by a change in self-control strategies, going back to working from the organization’s premises, personal demographics or job characteristics. Notably, this includes the change in the share of WFH between wave 1 and wave 3, satisfaction with working from home, working hours, self-control strategies such as goal reminders removing distractions from the workplace, frequency of communication with supervisors and colleagues, intrinsic work motivation and perceived organizational support. Controlling for any of these (and further) variables did not affect the positive adjustment of low self-control individuals and thus does not seem to be its driver. Thus, although our analysis did not reveal the mechanism behind the faster adjustment of low self-control individuals, we could rule out many potential explanations pertaining to the control variables listed above. We speculate that low self-control individuals might be less likely to set unattainable goal and have more goal flexibility compared to high self-control individuals–an ability that could be particularly beneficial in times of crisis [27, 42]. We hope that future studies will test these possibilities.

## Conclusion

Despite the bad reputation of low trait self-control in the workplace, we have shown that low self-control workers experienced a positive adjustment during the pandemic-induced transition to WFH, as evidenced by rising performance and decreasing work distractions. High self-control workers, on the other hand, did not change their performance significantly over time, remaining at a high-performance level throughout the study. As the shift to WFH is likely to be long-lasting [1], this represents an important step in documenting the factors that contribute to a successful WFH transition.

## Supporting information

S1 File Supplementary material. Additional analyses mentioned are presented in the supplementary material.
(PDF)
