# Supplementary material for: Self-control and performance while working from home
Source: PLoS One. 2023 Apr 13;18(4):e0282862. doi: 10.1371/journal.pone.0282862 (PMC10101465; doi:10.1371/journal.pone.0282862)
Supplement: S1 File — Additional analyses mentioned are presented in the supplementary material. (PDF) [file pone.0282862.s001.pdf]

Self-control and performance while working from home

SUPPLEMENTARY MATERIAL

Julia Baumann[a], Anastasia Danilov[a], & Olga Stavrova[b]

[a] Humboldt-Universität zu Berlin

[b] Tilburg University

S1 Additional Analyses

Correlation between the share of working from home and work distractions

Table S1: Share WFH does not predict work distractions

	Work distractions	
	**Model 1**	**Model 2**
Intercept	1.722*** [1.617, 1.828]	1.972*** [1.742, 2.202]
	(0.054)	(0.117)
% working from home	0.001 [0.000, 0.002]	0.001 [0.000, 0.002]
	(0.001)	(0.001)
Gender		0.017 [-0.061, 0.096]
Age		-0.012*** [-0.016, -0.008]
Children		0.120** [0.036, 0.204]
Income		0.010 [-0.011, 0.032]
Education		0.014 [-0.013, 0.041]
Num.Obs.	516	500
R2	0.003	0.098
R2 Adj.	0.001	0.087
AIC	642.6	566.3
BIC	655.3	600.1
Log.Lik.	-318.290	-275.170
F	1.707	8.906

Note: Results obtained from a pooled OLS model. 95% confidence intervals are in brackets. Significance levels are depicted as follows: *p<.05, **p<.01, ***p<0.001. Gender: 1 = female, 0 = male; Children: 0 = no minor children living in the household, 1 = at least one minor child living in the household.

Self-control*time interaction effect with depression and job satisfaction as outcome variables

Table S2 presents the same analyses as shown in Table 4 in the manuscript, but with depression and job satisfaction as outcome variables. Neither the effect of time trend, nor its

interaction with self-control were significant, suggesting that there were no temporal changes in the outcome variables on average and that this pattern did not depend on individuals' initial self-control level.

Table S2: Self-control and time trend predicting employee outcomes

	Depression		Jobs satisfaction	
	Model 1	Model 2	Model 1	Model 2
Intercept	-0.002 [-0.106, 0.103]	0.345 [-0.126, 0.816]	0.012 [-0.100, 0.123]	-0.329 [-0.944, 0.286]
Time	0.000 [-0.004, 0.005]	0.000 [-0.005, 0.005]	-0.002 [-0.008, 0.004]	-0.003 [-0.009, 0.003]
Self-control	-0.386*** [-0.491, -0.282]	-0.095 [-0.195, 0.005]	0.212*** [0.101, 0.323]	0.091 [-0.042, 0.225]
Time*Self-control	-0.001 [-0.006, 0.003]	-0.001 [-0.006, 0.003]	0.003 [-0.003, 0.008]	0.003 [-0.003, 0.008]
Gender		-0.193* [-0.379, -0.007]		0.275* [0.032, 0.519]
Age		-0.007 [-0.015, 0.002]		0.001 [-0.009, 0.012]
Children		-0.052 [-0.235, 0.130]		0.252* [0.014, 0.491]
Income		-0.002 [-0.049, 0.044]		0.015 [-0.046, 0.076]
Education		0.007 [-0.052, 0.066]		0.001 [-0.076, 0.078]
% working from home		-0.005 [-0.062, 0.051]		-0.030 [-0.099, 0.039]
Extraversion		-0.066 [-0.158, 0.027]		0.104 [-0.017, 0.225]
Agreeableness		0.044 [-0.047, 0.135]		-0.048 [-0.166, 0.071]
Conscientiousness		-0.140** [-0.234, -0.047]		0.045 [-0.077, 0.167]
Neuroticism		0.575*** [0.480, 0.670]		-0.255*** [-0.379, -0.131]
Openness		0.050 [-0.036, 0.137]		0.014 [-0.099, 0.127]
AIC	1590.5	1482.9	1768.7	1765.8
BIC	1627.8	1570.7	1805.9	1853.6
Log.Lik.	-787.272	-722.470	-876.355	-863.915
REMLcrit	1574.545	1444.939	1752.709	1727.829

Note: Results obtained from a linear mixed effects model with individual intercepts and slopes. 95% confidence intervals are in brackets. Significance levels are depicted as follows: *p<.05, **p<.01, ***p<0.001. Gender: 1 = female, 0 = male; Children: 0 = no minor children living in the household, 1 = at least one minor child living in the household.

Effect of self-control on employee outcomes at different time points

Table S3 shows that the effect of self-control on performance and work distractions diminishes over time but never turns non-significant, suggesting that even at wave 3, individuals with higher self-control still report higher performance and less work distractions than individuals with lower self-control.

Table S3: Effect of self-control on performance and work distractions at different points in time

	Performance			Work distractions	
	Wave 1	Wave 2	Wave 3	Wave 1	Wave 3
Intercept	0.000 [-0.115, 0.115]	0.000 [-0.117, 0.117]	0.000 [-0.120, 0.120]	0.000 [-0.114, 0.114]	0.000 [-0.119, 0.119]
Self-control	0.345*** [0.230, 0.461]	0.316*** [0.199, 0.432]	0.199** [0.079, 0.320]	-0.371*** [-0.485, -0.256]	-0.252*** [-0.372, -0.133]
Num.Obs.	258	258	258	258	258
R2	0.119	0.100	0.040	0.137	0.064
R2 Adj.	0.116	0.096	0.036	0.134	0.060
AIC	704.4	710.1	726.7	699.0	720.2
BIC	715.1	720.8	737.4	709.7	730.8
Log.Lik.	-349.211	-352.051	-360.353	-346.505	-357.087
F	34.648	28.319	10.596	40.808	17.432

Note: Results obtained from an Ordinary Least Squares regression. 95% confidence intervals are in brackets. Significance levels are depicted as follows: *p<.05, **p<.01, ***p<0.001.

Adding further control variables

Besides the control variables reported in the manuscript, the survey included further socio-demographics and work-related variables: Share of WFH pre-pandemic, WFH satisfaction, whether and how much they worked from home due to Covid-19, contractual number of working hours, actual number of working hours, number of days working per week, satisfaction working from organization’s premises, use of self-control strategies (Remove distractions, goal reminders, promise giving in after work, remind why distraction is bad, using willpower, giving in to distraction), jobs tasks (intellectual vs. manual, routine vs. creative, job independence), whether one was a supervisor, frequency of communication with colleagues and supervisors, intrinsic work motivation and perceived organizational support. With performance as the outcome variable, we also controlled for work distractions. Here, we tested whether including these variables into our main analyses (reported in Table 4 in the manuscript) changes the time x self-control interaction in any way. The results showed that the time x self-control interaction effect remains robust against adding all these control variables. In the following tables (Table S4 – Table S15), we present the obtained regression coefficients. All regression models reported here included the demographic and personality trait control variables that were also part of the main analyses reported in Table 4 (Gender, Age, Children, Income, Education, % working from home, Extraversion, Agreeableness, Conscientiousness, Neuroticism, Openness; not shown here to improve readability). Unless otherwise noted, N = 250.

*Table S4: Self-control*time interaction effect, when controlling for WFH characteristics (see table note for details); Outcome: Performance*

	Performance				
	Model 1	Model 2	Model 3	Model 4	Model 4
Intercept	0.069 [-0.477, 0.614]	0.081 [-0.465, 0.627]	0.036 [-0.510, 0.583]	0.042 [-0.475, 0.560]	0.004 [-0.575,

	Performance				
	Model 1	Model 2	Model 3	Model 4	Model 4
					0.583]
Self-control	0.272*** [0.153, 0.391]	0.274*** [0.155, 0.393]	0.269*** [0.150, 0.387]	0.256*** [0.143, 0.369]	0.270*** [0.151, 0.389]
Time	0.011*** [0.004, 0.017]	0.010** [0.004, 0.017]	0.010** [0.004, 0.017]	0.009** [0.003, 0.015]	0.011*** [0.005, 0.017]
**Time × self-control**	**-0.008* [-0.013, -0.002]**	**-0.008* [-0.013, -0.002]**	**-0.008* [-0.013, -0.002]**	**-0.008* [-0.013, -0.002]**	**-0.007* [-0.013, -0.002]**
**Difference in % WFH w3-w1**		**0.046 [-0.052, 0.144]**			
**% WFH pre-pandemic**			**0.002 [-0.001, 0.006]**		
**WFH satisfaction**				**0.212*** [0.135, 0.289]**	
**WFH due to COVID-19**					**0.022 [-0.043, 0.086]**
AIC	1928.2	1933.5	1939.2	1906.9	1934.7
BIC	2016.0	2025.9	2031.6	1999.3	2027.1
Log.Lik.	-945.088	-946.736	-949.607	-933.430	-947.367
REMLcrit	1890.175	1893.471	1899.214	1866.861	1894.735

Note: Results obtained from a linear mixed effects model with individual intercepts and slopes. 95% confidence intervals are in brackets. Significance levels are depicted as follows: *p<.05, **p<.01, ***p<0.001. Further controls included in this model were: Gender, Age, Children, Income, Education, % working from home, Big Five. Difference in % WFH w3-w1: % working from home in wave 3 minus the share working from home in wave 1. % WFH pre-pandemic: What share of your working hours did you use to work from home before the Covid-19 pandemic? 0-100. WFH satisfaction: How satisfied are you currently working from home? (Wave 1; Scale: 1-10). WFH due to COVID-19: “During the COVID-19 pandemic, are you working from home?” 1 = Yes, I am working from home every day. I rarely worked from home before COVID-19 (less than 1 day a week). 2 = Yes, I am working from home every day. I sometimes worked from home before COVID-19 (1 day a week or more). 3 = Yes, I am working from home every day. I always worked from home before COVID-19. 4 = Yes, I am sometimes working from home, but still commuting to my workplace on other days. 5 = No, I am still commuting to work every day, even during the COVID-19 pandemic. 6 = Due to the COVID-

19 outbreak, I am temporarily unemployed or not working (e.g. furloughed). 7 = I am currently unemployed or out of work, but not directly because of the COVID-19 outbreak. 8 = Other. 9 = Not applicable / rather not say.

*Table S5: Self-control*time interaction effect, when controlling for Job characteristics (see table note for details); Outcome: Performance*

	Performance			
	Model 1	Model 2	Model 3	Model 4
Intercept	0.255 [-0.558, 1.068]	-0.122 [-0.778, 0.533]	0.275 [-0.710, 1.260]	-0.302 [-1.275, 0.672]
Self-control	0.270*** [0.151, 0.389]	0.273*** [0.154, 0.392]	0.271*** [0.152, 0.390]	0.194* [0.044, 0.344]
Time	0.011*** [0.004, 0.017]	0.011*** [0.004, 0.017]	0.011*** [0.004, 0.017]	0.009 [0.000, 0.019]
**Time × self-control**	**-0.008* [-0.013, -0.002]**	**-0.007* [-0.013, -0.002]**	**-0.008* [-0.013, -0.002]**	**-0.006 [-0.014, 0.003]**
**Contractual working hours**	**-0.005 [-0.020, 0.011]**			
**Actual working hours**		**0.005 [-0.005, 0.016]**		
**Number of work days per week**			**-0.041 [-0.204, 0.122]**	
**Satisfaction performance at organization's premises**				**0.134 [-0.028, 0.297]**
AIC	1937.7	1937.8	1933.1	1025.4
BIC	2030.1	2030.2	2025.5	1104.8
Log.Lik.	-948.838	-948.888	-946.536	-492.681
REMLcrit	1897.675	1897.776	1893.072	985.361

Note: Results obtained from a linear mixed effects model with individual intercepts and slopes. 95% confidence intervals are in brackets. Significance levels are depicted as follows: *p<.05, **p<.01, ***p<0.001. Further controls included in this model were: Gender, Age, Children, Income, Education, % working from home, Big Five. Satisfaction performance at organization's premises: "How satisfied are you currently with your work performance working from your organization's premises?" (Scale: 1-5). In Model 4, N =131 due to the fact that some participants never worked from their organization's premises.

*Table S6: Self-control*time interaction effect, when controlling for use of self-control strategies (see table note for details); Outcome: Performance*

	Performance					
	Model 1	Model 2	Model 3	Model 4	Model 5	Model 6
Intercept	-0.049 [-0.636, 0.538]	-0.147 [-0.731, 0.437]	-0.023 [-0.603, 0.558]	-0.039 [-0.616, 0.538]	-0.303 [-0.882, 0.276]	0.741* [0.122, 1.360]
Self-control	0.257*** [0.135, 0.379]	0.253*** [0.134, 0.373]	0.266*** [0.146, 0.386]	0.259*** [0.138, 0.380]	0.223*** [0.102, 0.343]	0.209*** [0.089, 0.328]
Time	0.011*** [0.004, 0.017]	0.011*** [0.004, 0.017]	0.011*** [0.004, 0.017]	0.011*** [0.004, 0.017]	0.010** [0.004, 0.017]	0.011*** [0.004, 0.017]
**Time × self-control**	**-0.008* [-0.013, -0.002]**	**-0.008* [-0.013, -0.002]**	**-0.008* [-0.013, -0.002]**	**-0.008* [-0.013, -0.002]**	**-0.008* [-0.013, -0.002]**	**-0.008* [-0.013, -0.002]**
**Remove distractions**	**0.045 [-0.038, 0.128]**					
**Remind of goals**		**0.090* [0.000, 0.179]**				
**Promise giving in after work**			**0.034 [-0.040, 0.109]**			
**Remind why distraction is bad**				**0.043 [-0.033, 0.120]**		
**Using willpower**					**0.146** [0.059, 0.233]**	
**Giving in to distraction**						**-0.243*** [-0.360, -0.127]**
AIC	1933.5	1930.7	1934.1	1933.6	1924.0	1917.7
BIC	2025.9	2023.1	2026.5	2026.0	2016.4	2010.1
Log.Lik.	-946.768	-945.331	-947.032	-946.792	-941.981	-938.862
REMLcrit	1893.536	1890.661	1894.063	1893.585	1883.961	1877.723

Note: Results obtained from a linear mixed effects model with individual intercepts and slopes. 95% confidence intervals are in brackets. Significance levels are depicted as follows: *p<.05, **p<.01, ***p<0.001. Further controls included in this model were: Gender, Age, Children, Income, Education, % working from home, Big Five. Self-control strategies: “Think of your working from home during the past seven days. How

often did you follow the behaviors described below when you felt distracted?” I removed any distractions from my workspace before starting to work; I reminded myself of my work goals when I got distracted; I made myself a promise to give into distraction later after I was done with my work; I reminded myself of why it was bad for me to let myself get distracted; I simply resisted the distraction; I gave in to the distraction. (Scale: Never (1) to Always (5)). Remove distractions

*Table S7: Self-control*time interaction effect, when controlling for work tasks (see table note for details); Outcome: Performance*

	Performance		
	Model 1	Model 2	Model 3
Intercept	-0.128 [-0.740, 0.485]	0.091 [-0.462, 0.643]	-0.335 [-0.965, 0.294]
Self-control	0.263*** [0.143, 0.382]	0.274*** [0.155, 0.393]	0.260*** [0.142, 0.378]
Time	0.011*** [0.004, 0.017]	0.011*** [0.004, 0.017]	0.011*** [0.004, 0.017]
**Time × self-control**	**-0.008* [-0.013, -0.002]**	**-0.008* [-0.013, -0.002]**	**-0.007* [-0.013, -0.002]**
**Manual/intellectual tasks**	**0.036 [-0.015, 0.087]**		
**Routine/creative tasks**		**-0.011 [-0.051, 0.029]**	
**Job independence**			**0.063* [0.013, 0.114]**
AIC	1933.7	1935.9	1929.7
BIC	2026.1	2028.3	2022.1
Log.Lik.	-946.866	-947.929	-944.841
REMLcrit	1893.732	1895.857	1889.682

Note: Results obtained from a linear mixed effects model with individual intercepts and slopes. 95% confidence intervals are in brackets. Significance levels are depicted as follows: *p<.05, **p<.01, ***p<0.001. Further controls included in this model were: Gender, Age, Children, Income, Education, % working from home, Big Five. Manual/intellectual tasks: Are the tasks you do at work rather manual or intellectual? (Scale: 0-10). Routine/creative tasks: “Are the tasks you perform at work rather routine tasks or creative tasks?” (Scale: 0-10). Job independence: “How much independence do you have in performing your tasks at work?” (Scale: 0-10).

*Table S8: Self-control*time interaction effect, when controlling for supervising and communication with supervisors and colleagues (see table note for details); Outcome: Performance*

	Performance		
	Model 1	Model 2	Model 3
Intercept	0.409 [-0.307, 1.124]	0.122 [-0.588, 0.831]	0.301 [-0.525, 1.127]
Self-control	0.267*** [0.148, 0.386]	0.272*** [0.153, 0.391]	0.274*** [0.155, 0.393]
Time	0.011*** [0.005, 0.017]	0.011*** [0.004, 0.017]	0.011*** [0.004, 0.017]
**Time × self-control**	**-0.007* [-0.013, -0.002]**	**-0.008* [-0.013, -0.002]**	**-0.007* [-0.013, -0.002]**
**Supervisor**	**-0.147 [-0.348, 0.054]**		
**Communication with supervisor**		**-0.013 [-0.122, 0.096]**	
**Communication with colleagues**			**-0.061 [-0.224, 0.102]**
AIC	1930.8	1934.1	1932.8
BIC	2023.2	2026.5	2025.2
Log.Lik.	-945.419	-947.035	-946.392
REMLcrit	1890.839	1894.070	1892.783

Note: Results obtained from a linear mixed effects model with individual intercepts and slopes. 95% confidence intervals are in brackets. Significance levels are depicted as follows: *p<.05, **p<.01, ***p<0.001. Further controls included in this model were: Gender, Age, Children, Income, Education, % working from home, Big Five. Supervisor: “Do you supervise other people at work?” 0 = No; 1 = Yes. Communication with supervisor/Communication with colleagues: “On average, how often do you communicate with your direct supervisor?/your colleagues?”: 4 = Daily; 5 = Weekly; 6 = Monthly; 7 = Less than monthly; 8 = Does not apply.

*Table S9: Self-control*time interaction effect, when controlling intrinsic work motivation & perceived organizational support and work distractions (see table note for details); Outcome: Performance*

Performance

	Model 1	Model 2	Model 3
Intercept	-0.025 [-0.623, 0.574]	-0.334 [-0.983, 0.315]	0.375 [-0.182, 0.933]
Self-control	0.263*** [0.142, 0.384]	0.257*** [0.138, 0.376]	0.005 [-0.002, 0.012]
Time	0.011*** [0.004, 0.017]	0.011*** [0.004, 0.017]	0.281*** [0.167, 0.395]
**Time × self-control**	**-0.008* [-0.013, -0.002]**	**-0.008* [-0.013, -0.002]**	**-0.007* [-0.014, -0.001]**
**Intrinsic work motivation**	**0.036 [-0.058, 0.130]**		
**Perceived org. support**		**0.084* [0.009, 0.158]**	
**Work distractions**			**-0.249*** [-0.339, -0.158]**
AIC	1933.9	1930.0	
BIC	2026.3	2022.4	
Log.Lik.	-946.925	-945.006	
REMLcrit	1893.850	1890.013	

Note: Results obtained from a linear mixed effects model with individual intercepts and slopes. 95% confidence intervals are in brackets. Significance levels are depicted as follows: *p<.05, **p<.01, ***p<0.001. Further controls included in this model were: Gender, Age, Children, Income, Education, % working from home, Big Five. Intrinsic work motivation: 3-item Intrinsic motivation sub-scale by Tremblay et al. (2009) (5-point Likert scale); Perceived org. support: Perceived organizational support measured with the short 8-item scale by Eisenberger et al. (1986) (7-point Likert scale); Work distractions are defined as an index of different sources of distractions as described in the paper.

*Table S10: Self-control*time interaction effect, when controlling for WFH characteristics (see table note for details); Outcome: Work distractions*

	Work distractions				
	Model 1	Model 2	Model 3	Model 4	Model 4
Intercept	0.518 [-0.061, 1.097]	0.500 [-0.078, 1.077]	0.491 [-0.090, 1.072]	0.539 [-0.023, 1.100]	0.656* [0.041, 1.271]
Self-control	-0.312*** [-0.443, -0.181]	-0.315*** [-0.446, -0.184]	-0.314*** [-0.445, -0.183]	-0.301*** [-0.429, -0.173]	-0.309*** [-0.440, -0.178]
Time	-0.009** [-0.015, -0.003]	-0.008** [-0.014, -0.002]	-0.009** [-0.015, -0.003]	-0.008* [-0.014, -0.002]	-0.010** [-0.016, -0.004]
**Time × self-**	**0.007* [0.001,**	**0.007* [0.001,**	**0.007* [0.001,**	**0.007* [0.001,**	

	Work distractions				
	Model 1	Model 2	Model 3	Model 4	Model 4
**control**	**0.012]**	**0.012]**	**0.012]**	**0.012]**	**0.006* [0.001, 0.012]**
**Difference in % WFH w3-w1**		**-0.080 [-0.188, 0.027]**			
**% WFH pre-pandemic**			**0.002 [-0.002, 0.006]**		
**WFH satisfaction**				**-0.130** [-0.214, -0.045]**	
**WFH due to COVID-19**					**-0.047 [-0.118, 0.025]**
AIC	1268.0	1271.8	1279.6	1265.9	1273.1
BIC	1339.6	1347.7	1355.4	1341.8	1349.0
Log.Lik.	-616.998	-617.901	-621.793	-614.963	-618.570
REMLcrit	1233.996	1235.803	1243.585	1229.926	1237.140

Note: Results obtained from a linear mixed effects model with individual intercepts and slopes. 95% confidence intervals are in brackets. Significance levels are depicted as follows: *p<.05, **p<.01, ***p<0.001. Further controls included in this model were: Gender, Age, Children, Income, Education, % working from home, Big Five. Difference in % WFH w3-w1: % working from home in wave 3 minus the share working from home in wave 1. WFH satisfaction: How satisfied are you currently working from home? (Wave 1; Scale: 1-10). WFH due to COVID-19: “During the COVID-19 pandemic, are you working from home?” 1 = Yes, I am working from home every day. I rarely worked from home before COVID-19 (less than 1 day a week). 2 = Yes, I am working from home every day. I sometimes worked from home before COVID-19 (1 day a week or more). 3 = Yes, I am working from home every day. I always worked from home before COVID-19. 4 = Yes, I am sometimes working from home, but still commuting to my workplace on other days. 5 = No, I am still commuting to work every day, even during the COVID-19 pandemic. 6 = Due to the COVID-19 outbreak, I am temporarily unemployed or not working (e.g. furloughed). 7 = I am currently unemployed or out of work, but not directly because of the COVID-19 outbreak. 8 = Other. 9 = Not applicable / rather not say.

*Table S11: Self-control*time interaction effect, when controlling for job characteristics (see table note for details); Outcome: Work distractions*

Work distractions				
	Model 1	Model 2	Model 3	Model 4
Intercept	0.477 [-0.386, 1.340]	0.444 [-0.253, 1.141]	0.414 [-0.631, 1.458]	0.432 [-0.694, 1.559]
Self-control	-0.311*** [-0.443, -0.180]	-0.311*** [-0.442, -0.180]	-0.311*** [-0.442, -0.180]	-0.235* [-0.422, -0.049]
Time	-0.009** [-0.015, -0.003]	-0.009** [-0.015, -0.003]	-0.009** [-0.015, -0.003]	-0.006 [-0.017, 0.005]
**Time × self-control**	**0.007* [0.001, 0.012]**	**0.007* [0.001, 0.012]**	**0.007* [0.001, 0.012]**	**0.008 [-0.001, 0.018]**
**Contractual working hours**	**0.001 [-0.015, 0.017]**			
**Actual working hours**		**0.002 [-0.009, 0.013]**		
**Number of work days per week**			**0.021 [-0.152, 0.194]**	
**Satisfaction performance at organization's premises**				**0.074 [-0.115, 0.263]**
AIC	1277.7	1278.4	1273.0	735.2
BIC	1353.6	1354.3	1348.8	799.4
Log.Lik.	-620.866	-621.203	-618.484	-349.601
REMLcrit	1241.731	1242.406	1236.967	699.202

Note: Results obtained from a linear mixed effects model with individual intercepts and slopes. 95% confidence intervals are in brackets. Significance levels are depicted as follows: *p<.05, **p<.01, ***p<0.001. Further controls included in this model were: Gender, Age, Children, Income, Education, % working from home, Big Five. Satisfaction performance at organization's premises: "How satisfied are you currently with your work performance working from your organization's premises?" (Scale: 1-5). In Model 4, N =131 due to the fact that some participants never worked from their organization's premises.

*Table S12 Self-control*time interaction effect, when controlling for use of self-control strategies (see table note for details); Outcome: Work distractions*

Work distractions						
	Model 1	Model 2	Model 3	Model 4	Model 5	Model 6
Intercept	0.802*	0.722*	0.680*	0.469 [-	1.018**	-0.641* [-

Work distractions						
	Model 1	Model 2	Model 3	Model 4	Model 5	Model 6
	[0.186, 1.419]	[0.102, 1.343]	[0.066, 1.294]	0.145, 1.083]	[0.412, 1.624]	1.258, -0.024]
Self-control	-0.276*** [-0.409, -0.143]	-0.294*** [-0.426, -0.162]	-0.301*** [-0.433, -0.170]	-0.318*** [-0.451, -0.184]	-0.246*** [-0.376, -0.115]	-0.203** [-0.327, -0.078]
Time	-0.009** [-0.015, -0.003]	-0.009** [-0.015, -0.003]	-0.009** [-0.015, -0.003]	-0.009** [-0.015, -0.003]	-0.009** [-0.015, -0.003]	-0.009** [-0.015, -0.003]
**Time × self-control**	**0.007* [0.001, 0.012]**	**0.007* [0.001, 0.012]**	**0.007* [0.001, 0.012]**	**0.007* [0.001, 0.012]**	**0.007* [0.001, 0.012]**	**0.007* [0.001, 0.012]**
**Remove distractions**	**-0.109* [-0.196, -0.021]**					
		**-0.085 [-0.180, 0.010]**				
**Remind of goals**			**-0.061 [-0.139, 0.018]**			
**Promise giving in after work**				**0.020 [-0.061, 0.101]**		
**Remind why distraction is bad**					**-0.196*** [-0.287, -0.105]**	
**Using willpower**						**0.420*** [0.304, 0.537]**
AIC	1268.4	1271.2	1272.3	1274.3	1256.9	1227.7
BIC	1344.3	1347.0	1348.2	1350.2	1332.8	1303.6
Log.Lik.	-616.214	-617.576	-618.149	-619.155	-610.461	-595.855
REMLcrit	1232.427	1235.152	1236.299	1238.310	1220.921	1191.710

Note: Results obtained from a linear mixed effects model with individual intercepts and slopes. 95% confidence intervals are in brackets. Significance levels are depicted as follows: *p<.05, **p<.01, ***p<0.001. Further controls included in this model were: Gender, Age, Children, Income, Education, % working from home, Big Five. Self-control strategies: “Think of your working from home during the past seven days. How often did you follow the behaviors described below when you felt distracted?” I removed any distractions from my workspace before starting to work; I reminded myself of my work goals when I got distracted; I made

myself a promise to give into distraction later after I was done with my work; I reminded myself of why it was bad for me to let myself get distracted; I simply resisted the distraction (Scale: Never (1) to Always (5)).

*Table S13: Self-control*time interaction effect, when controlling for work tasks (see table note for details); Outcome: Work distractions*

	Work distractions		
	Model 1	Model 2	Model 3
Intercept	0.670* [0.018, 1.321]	0.476 [-0.110, 1.061]	0.888** [0.218, 1.558]
Self-control	-0.305*** [-0.436, -0.173]	-0.316*** [-0.447, -0.184]	-0.301*** [-0.431, -0.170]
Time	-0.009** [-0.015, -0.003]	-0.009** [-0.015, -0.003]	-0.009** [-0.015, -0.003]
**Time × self-control**	**0.007* [0.001, 0.012]**	**0.007* [0.001, 0.012]**	**0.007* [0.001, 0.012]**
**Manual/intellectual tasks**	**-0.028 [-0.082, 0.027]**		
**Routine/creative tasks**		**0.020 [-0.022, 0.063]**	
**Job independence**			**-0.058* [-0.111, -0.004]**
AIC	1274.3	1274.9	1270.9
BIC	1350.2	1350.8	1346.8
Log.Lik.	-619.167	-619.465	-617.465
REMLcrit	1238.335	1238.930	1234.929

Note: Results obtained from a linear mixed effects model with individual intercepts and slopes. 95% confidence intervals are in brackets. Significance levels are depicted as follows: *p<.05, **p<.01, ***p<0.001. Further controls included in this model were: Gender, Age, Children, Income, Education, % working from home, Big Five. Manual/intellectual tasks: Are the tasks you do at work rather manual or intellectual? (Scale: 0-10). Routine/creative tasks: “Are the tasks you perform at work rather routine tasks or creative tasks?” (Scale: 0-10). Job independence: “How much independence do you have in performing your tasks at work?” (Scale: 0-10).

*Table S14: Self-control*time interaction effect, when controlling for supervising, communication with supervisors and colleagues (see table note for details); Outcome: Work distractions*

Work distractions

	Model 1	Model 2	Model 3
Intercept	0.597 [-0.165, 1.359]	0.667 [-0.086, 1.420]	0.553 [-0.325, 1.431]
Self-control	-0.313*** [-0.444, -0.181]	-0.312*** [-0.443, -0.180]	-0.311*** [-0.443, -0.180]
Time	-0.009** [-0.015, -0.003]	-0.009** [-0.015, -0.003]	-0.009** [-0.015, -0.003]
**Time × self-control**	**0.007* [0.001, 0.012]**	**0.007* [0.001, 0.012]**	**0.007* [0.001, 0.012]**
**Supervisor**	**-0.034 [-0.248, 0.180]**		
**Communication with supervisor**		**-0.036 [-0.152, 0.080]**	
**Communication with colleagues**			**-0.009 [-0.182, 0.164]**
AIC	1272.5	1273.5	1273.0
BIC	1348.4	1349.3	1348.9
Log.Lik.	-618.251	-618.726	-618.505
REMLcrit	1236.502	1237.453	1237.010

Note: Results obtained from a linear mixed effects model with individual intercepts and slopes. 95% confidence intervals are in brackets. Significance levels are depicted as follows: *p<.05, **p<.01, ***p<0.001. Further controls included in this model were: Gender, Age, Children, Income, Education, % working from home, Big Five. Supervisor: "Do you supervise other people at work?" 0 = No; 1 = Yes. Communication with supervisor/Communication with colleagues: "On average, how often do you communicate with your direct supervisor?/your colleagues?": 4 = Daily; 5 = Weekly; 6 = Monthly; 7 = Less than monthly; 8 = Does not apply.

*Table S15: Self-control*time interaction effect, when controlling for intrinsic work motivation & perceived organizational support (see table note for details); Outcome: Work distractions*

	Work distractions	
	Model 1	Model 2
Intercept	0.329 [-0.303, 0.962]	0.441 [-0.255, 1.136]
Self-control	-0.330*** [-0.463, -0.197]	-0.314*** [-0.446, -0.182]
Time	-0.009** [-0.015, -0.003]	-0.009** [-0.015, -0.003]
**Time × self-control**	**0.007* [0.001, 0.012]**	**0.007* [0.001, 0.012]**
**Intrinsic work motivation**	**0.073 [-0.027, 0.173]**	
**Perceived org. support**		**0.016 [-0.064, 0.096]**
AIC	1272.0	1274.4
BIC	1347.9	1350.3

	Work distractions	
	Model 1	Model 2
Log.Lik.	-618.019	-619.208
REMLcrit	1236.039	1238.415

Note: Results obtained from a linear mixed effects model with individual intercepts and slopes. 95% confidence intervals are in brackets. Significance levels are depicted as follows: *p<.05, **p<.01, ***p<0.001. Gender: 1 = female, 0 = male; Children: 0 = no minor children living in the household, 1 = at least one minor child living in the household. Intrinsic work motivation: 3-item Intrinsic motivation sub-scale by Tremblay et al. (2009) (5-point Likert scale); Perceived org. support: Perceived organizational support measured with the short 8-item scale by Eisenberger et al. (1986) (7-point Likert scale).

Depicting simple slopes for performance and work distractions for different levels of self-control

Table S16: Comparing simple slopes across different levels of self-control

**Levels of self-control**	*Min (-2.41 SD)*	*-2 SD*	*-1 SD*	*Mean*	*+ 1 SD*	*+ 2 SD*	*Max (+2.37 SD)*
	Performance						
**Estimate**	*0.029****	*0.026****	*0.018****	*0.010****	*0.002*	*-0.006*	*-0.009*
**t-statistic**	*3.757*	*3.886*	*4.221*	*3.249*	*0.373*	*-0.981*	*-1.244*
**p-value**	*<0.001*	*<0.001*	*<0.001*	*0.001*	*0.710*	*0.327*	*0.214*
	Work distractions						
**Estimate**	*-0.025****	*-0.022****	*-0.017****	*-0.011****	*-0.005*	*0.001*	*0.004*
**t-statistic**	*-3.429*	*-3.609*	*-4.192*	*-3.786*	*-1.160*	*0.225*	*0.503*
**p-value**	*0.001*	*<0.001*	*<0.001*	*<0.001*	*0.247*	*0.823*	*0.615*
	Well-being						
**Estimate**	-0.11	*-0.009*	*-0.004*	*0.000*	*0.005*	*0.009*	*0.011*
**t-statistic**	*-1.920*	*-1.856*	*-1.450*	*0.049*	*1.519*	*1.899*	*1.953*
**p-value**	*0.055*	*0.064*	*0.148*	*0.961*	*0.129*	*0.058*	*0.051*

Note: The table depicts the simple slopes based on the linear mixed effects model (Model 1 for each outcome) with individual intercepts and slopes in Table 4 in the main text. $^{*}p<.05$, $^{**}p<.01$, $^{***}p<0.001$.

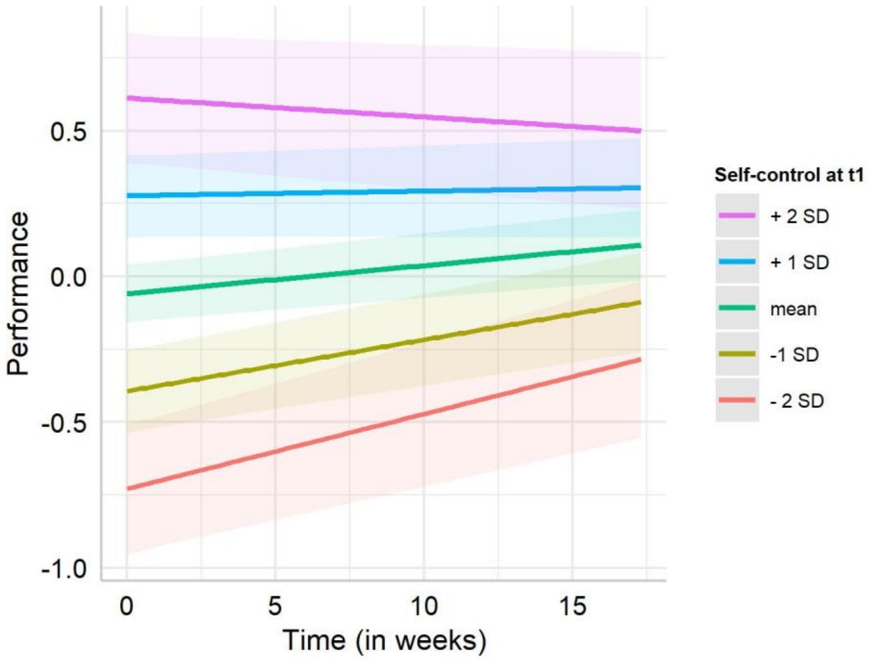

Fig S1: Predicted values of performance. Simple slope estimates for Model 1 (dependent variable: performance) in Table 4. The figure depicts predicted values of performance (standardized around 0) over time for low self-control (-1 SD and -2 SD below mean), average self-control and high self-control (+1 SD and +2 SD above mean) individuals.

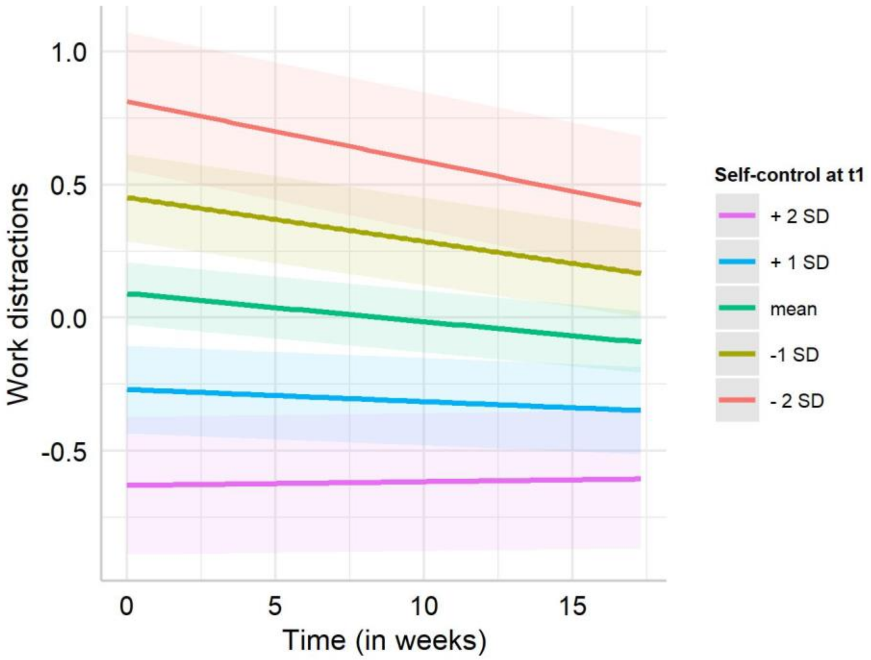

Fig S2: Predicted values of work distractions. Simple slope estimates for Model 1 (dependent variable: work distractions) in Table 4. The figure depicts predicted values of work distractions (standardized around 0) over time for low self-control (-1 SD and -2 SD below mean), average self-control and high self-control (+1 SD and +2 SD above mean) individuals.

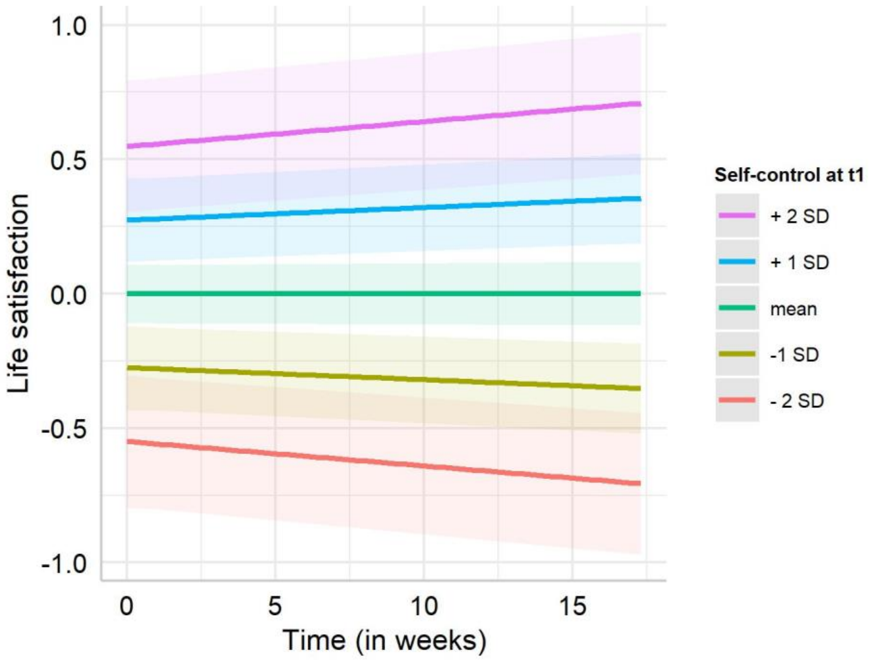

Fig S3: Predicted values of life satisfaction. Simple slope estimates for Model 1 (dependent variable: life satisfaction) in Table 4. The figure depicts predicted values of life satisfaction (standardized around 0) over time for low self-control (-1 SD and -2 SD below mean), average self-control and high self-control (+1 SD and +2 SD above mean) individuals.

Estimating a Tobit model for censored regressions with the performance measure as dependent variable

Table S17: Tobit model for censored regressions; outcome: performance

	**Performance**			
	Model 1 (LME)	Model 2 (LME)	Model 3 (Tobit)	Model 4 (Tobit)
Intercept	-0.059	0.077	0.065	0.066
Time	0.010**	0.011***	0.013***	0.015***
Self-control	0.335***	0.272***	0.402***	0.312***
Time*Self-control	-0.008**	-0.008*	-0.010*	-0.009*
Gender		0.274*		0.327*
Age		-0.004		-0.002
Children		-0.154		-0.193
Income		0.022		0.006
Education		-0.031		-0.012
% working from home		-0.007		0.008
Extraversion		0.011		0.053
Agreeableness		-0.012		-0.032
Conscientiousness		0.110*		0.153*
Neuroticism		-0.036		-0.035
Openness		0.065		0.076
Log Sigma Mu			-0.179**	-0.253***
Log Sigma Nu			-0.251***	-0.255***
REMLcrit	1953.5	1925.4		
AIC	1981.4	2003.9	2034.0	1954.2
BIC	-970.757	-945.689		
Log.Lik.	1941.514	1891.378	-1011.024	-960.090

Note: Model 1 and Model 2 replicate the main results in Table 4 in the main text, obtained from a linear mixed effects regression. Model 3 and Model 4 are obtained from a Tobit model with individual intercept and random slopes. 95% confidence intervals are in brackets. Significance levels are depicted as follows: *p<.05, **p<.01, ***p<0.001. Gender: 1 = female, 0 = male; Children: 0 = no minor children living in the household, 1 = at least one minor child living in the household.

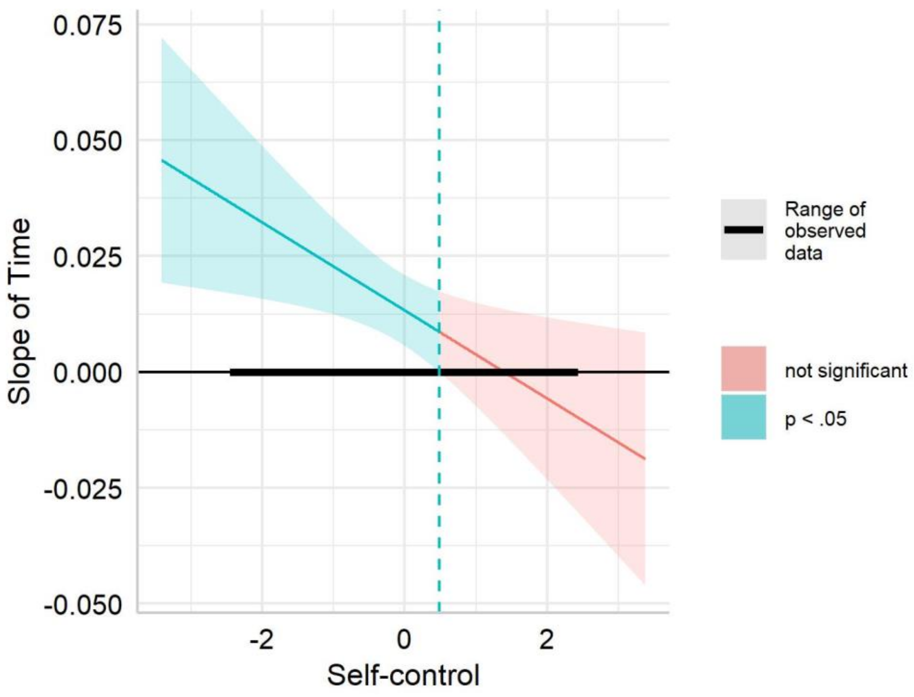

Fig S4: Johnson-Neyman intervals for performance based on a Tobit model. Johnson-Neyman intervals for the simple slope estimates for Model 3, S17 (dependent variable: performance) in Table 4. The figure depicts the estimated slope of time for each level of trait self-control (standardized around 0) and highlights for which values of trait self-control the simple slope estimate is significant.

Pre-pandemic share of WFH: Interaction with self-control and time

*Table S18: Share WFH pre-pandemic does not qualify the self-contol*time interaction*

	**Performance**	**Work distractions**	**Life satisfaction**
Intercept	-0.055 [-0.156, 0.046]	0.091 [-0.026, 0.207]	0.001 [-0.110, 0.112]
% WFH pre-pandemic	0.081 [-0.021, 0.182]	-0.033 [-0.150, 0.084]	0.035 [-0.076, 0.147]
Self-control	0.337*** [0.236, 0.438]	-0.362*** [-0.478, -0.245]	0.275*** [0.164, 0.386]
Time	0.010** [0.004, 0.015]	-0.011*** [-0.016, -0.005]	0.000 [-0.004, 0.005]
%WFHpre-pandemic* Self-control	0.069 [-0.026, 0.163]	-0.013 [-0.121, 0.096]	0.032 [-0.071, 0.135]
%WFHpre-pandemic* Time	-0.003 [-0.009, 0.003]	-0.002 [-0.007, 0.004]	0.002 [-0.003, 0.006]
Time*Self-control	-0.008** [-0.014, -0.002]	0.006* [0.000, 0.011]	0.005+ [0.000, 0.009]
**%WFHpre-pandemic* Time*Self-control**	**0.001 [-0.005, 0.006]**	**-0.003 [-0.008, 0.002]**	**0.001 [-0.003, 0.005]**
AIC	1987.4	1326.5	1726.6
BIC	2043.2	1368.9	1782.5
Log.Lik.	-981.700	-653.239	-851.320
REMLcrit	1963.399	1306.477	1702.641

Results obtained from a linear mixed effects model with individual intercepts and random slopes. 95% confidence intervals are in brackets. Significance levels are depicted as follows: +p<.1, *p<.05, **p<.01, ***p<0.001. Gender: 1 = female, 0 = male; Children: 0 = no minor children living in the household, 1 = at least one minor child living in the household.

S2 Details about the experimental intervention

Description of the experiment

With an experimental intervention, we investigated the impact of different self-control strategies on employees working from home. Specifically, we were interested in whether

randomly assigning participants to execute specific self-control strategies could have a positive impact on their productivity, goal achievement and well-being. The treatments consisted of selected behavioral strategies that had previously been shown to be associated with higher self-control Milyavskaya et al. (2020). The behavioral strategies we focused on were (see survey questions for exact instructions):

- Remove any distractions from your workplace. (Situation modification treatment)
- Remind yourself of your (work) goals. (Goal reminder treatment)
- When you face a distraction, promise yourself to give in later instead of now. (Give in later treatment)
- The control group was not given any specific strategy.

The initial sample size of 400 participants was chosen to be able to detect a small treatment effect (Cohen's f2) of 0.03. We contacted the subjects three times: First, for an in-take survey and treatment manipulation; second, after 3 days with a reminder of the treatment manipulation; and third after 7 days with a second survey. At the end of the first survey, participants were randomly assigned to one of the four experimental groups (three treatment groups plus one control group). In the treatments, subjects were asked to follow the respective strategy every day while working during the following week. They were also asked to write down a short text regarding how they plan to implement the respective strategy. In the middle of the week, we contacted subjects with a personalized reminder email. At the end of the week, participants were asked about their experiences during the experimental period in a second survey. We assessed the use of the assigned strategy and its success using the subjects’ (change in) self-reported productivity (performance), goal achievement and well-being.

Experimental design, hypotheses, and data analysis were pre-registered[1]. Our main hypothesis was that the treatment manipulations would increase productivity, goal achievement, and job satisfaction among the subjects in the treatment groups as compared to the control condition. With a chi-square test we tested whether individuals in each treatment groups reported using their assigned self-control strategy more often than individuals in other treatment groups. We found that only the “Situation modification” treatment group used their assigned strategy significantly more often than individuals assigned to other groups ($\chi^2(4) = 36.67$, $p < .001$). The treatment groups “Goal reminder” and “Give in later” did not report using their assigned strategy significantly more often than individuals in other groups ($\chi^2(4) = 2.77$, $p = .598$ and $\chi^2(4) = 6.90$, $p = .145$, respectively). Furthermore, in the “Give in Later” and “Goal reminder” treatments, only 69.9% (56/81) and 65.5% (57/87) of individuals reported the correct strategy when directly asked which strategy they had been asked to follow (80% (72/90) answered this correctly in the control group and 98.8% (83/84) in the “Situation modification” treatment). We thus conclude that the treatment manipulation did not work properly overall. In our analysis, we did not find any significant effects of the treatments on these pre-registered primary outcomes or (also pre-registered) secondary outcomes (depression, stress at work, focus, satisfaction with life in general and specific domains).

Table S19: Experimental Results - Primary Outcomes

	Dependent variable:			
	Performance	Job satisfaction	WFH Satisfaction	Goal achievement
	OLS	*OLS*	*OLS*	*linear mixed-effects*
	(1)	(2)	(3)	(5)

[1] Available in the AER Registry: AEARCTR-0007147.

Treatment Situation modification	0.149	-0.031	0.086	0.056
	(0.084)	(0.190)	(0.199)	(0.126)
Treatment Goal reminder	0.086	-0.169	-0.214	0.069
	(0.078)	(0.205)	(0.228)	(0.129)
Treatment Give in later	0.128	-0.305	0.046	-0.129
	(0.081)	(0.192)	(0.194)	(0.127)
Performance t1	0.533			
	(0.066)			
Job satisfaction t1		0.748		
		(0.040)		
WFH Satisfaction t1			0.823	
			(0.032)	
Goal achievement				
Constant	1.935*	1.959**	1.320***	3.738***
	(0.315)	(0.294)	(0.304)	(0.091)
Observations	258	258	258	774
Adjusted R^2	0.284	0.662	0.731	
Residual Std. Error	0.440 (df = 253)	1.204 (df = 253)	1.187 (df = 253)	
***Note**:*	Results obtained with an OLS regression with heterogeneity robust standard errors. P-values are in brackets. Significance levels are depicted as follows: *p<.05, **p<.01, ***p<0.001. Outcome variables were measured in t2, one week after the treatment manipulation in t1. Reference group is the control group.			

Self-control*time interaction effect when controlling for the experimental condition

We replicated our main findings (self-control x time interaction) reported in Table 4 in the manuscript when adding the experimental conditions (using dummy coding) as additional controls.

Table S20: Self-control and time trend predicting employee outcomes

	**Performance**		**Distraction score**		**Life satisfaction**	
	**Model 1**	**Model 2**	**Model 1**	**Model 2**	**Model 1**	**Model 2**
Intercept	-0.059 [-0.160, 0.043]	0.132 [-0.442, 0.707]	0.091 [-0.025, 0.207]	0.471 [-0.135, 1.078]	-0.001 [-0.111, 0.110]	-0.032 [-0.630, 0.565]
Time	0.010** [0.004, 0.015]	0.011*** [0.004, 0.017]	-0.011*** [-0.016, -0.005]	-0.009** [-0.015, -0.003]	0.000 [-0.005, 0.005]	-0.001 [-0.006, 0.004]

Table S20: Self-control and time trend predicting employee outcomes

	Performance		Distraction score		Life satisfaction	
	**Model 1**	**Model 2**	**Model 1**	**Model 2**	**Model 1**	**Model 2**
Self-control	0.335*** [0.234, 0.436]	0.274*** [0.154, 0.394]	-0.361*** [-0.477, -0.244]	-0.315*** [-0.446, -0.183]	0.274*** [0.164, 0.385]	0.075 [-0.049, 0.199]
**Time*Self-control**	**-0.008** [-0.014, -0.002]**	**-0.008* [-0.013, -0.002]**	**0.006* [0.000, 0.011]**	**0.007* [0.001, 0.012]**	**0.005 [0.000, 0.009]**	**0.004 [0.000, 0.009]**
Gender		0.271* [0.052, 0.490]		-0.114 [-0.345, 0.117]		0.281* [0.054, 0.509]
Age		-0.004 [-0.014, 0.005]		-0.020*** [-0.030, -0.010]		-0.002 [-0.012, 0.008]
Children		-0.164 [-0.377, 0.048]		0.264* [0.039, 0.488]		0.187 [-0.035, 0.408]
Income		0.021 [-0.033, 0.076]		0.012 [-0.045, 0.069]		0.032 [-0.025, 0.089]
Education		-0.028 [-0.096, 0.041]		0.045 [-0.027, 0.118]		-0.010 [-0.082, 0.062]
% working from home		-0.006 [-0.079, 0.067]		0.047 [-0.032, 0.127]		-0.023 [-0.085, 0.038]
Extraversion		0.018 [-0.090, 0.126]		0.134* [0.020, 0.248]		0.107 [-0.005, 0.220]
Agreeableness		-0.011 [-0.118, 0.096]		0.074 [-0.039, 0.187]		0.016 [-0.095, 0.127]
Conscientiousness		0.110* [0.001, 0.219]		0.010 [-0.106, 0.125]		0.083 [-0.031, 0.197]
Neuroticism		-0.037 [-0.148, 0.075]		0.180** [0.062, 0.298]		-0.384*** [-0.500, -0.267]
Openness		0.065 [-0.035, 0.166]		-0.014 [-0.120, 0.092]		-0.073 [-0.177, 0.032]
**Treatment Situation modification**		**-0.080 [-0.352, 0.192]**		**0.100 [-0.187, 0.386]**		**-0.175 [-0.457, 0.108]**
**Treament Give in Later**		**-0.108 [-0.379, 0.162]**		**0.098 [-0.188, 0.383]**		**-0.294* [-0.576, -0.012]**
**Treatment Goal reminder**		**-0.020 [-0.297, 0.257]**		**-0.113 [-0.405, 0.179]**		**-0.286 [-0.574, 0.003]**
AIC	1956.2	1940.5	1293.3	1277.9	1691.7	1664.7
BIC	1993.4	2042.1	1318.8	1362.2	1728.9	1766.4
Log.Lik.	-970.108	-948.228	-640.650	-618.967	-837.843	-810.374
REMLcrit	1940.216	1896.456	1281.300	1237.933	1675.685	1620.747

Results obtained from a linear mixed effects model with individual intercepts and random slopes. 95% confidence intervals are in brackets. Significance levels are depicted as follows: *p<.05, **p<.01, ***p<0.001. Gender: 1 = female, 0 = male; Children: 0 = no minor children living in the household, 1 = at least one minor child living in the household.

S3 Survey questions

Wave 1

What is your employment status?

- ○ Full-time employed
- ○ Part-time employed
- ○ Unemployed
- ○ Retired
- ○ Other
- ○ Homemaker
- ○ Student

In what country do you currently reside?

During the COVID-19 pandemic, are you working from home?

- ○ Yes, I am working from home every day. I rarely worked from home before COVID-19 (less than 1 day a week). (1)
- ○ Yes, I am working from home every day. I sometimes worked from home before COVID-19 (1 day a week or more). (2)
- ○ Yes, I am working from home every day. I always worked from home before COVID-19. (3)
- ○ Yes, I am sometimes working from home, but still commuting to my workplace on other days. (4)
- ○ No, I am still commuting to work every day, even during the COVID-19 pandemic. (5)

○ Due to the COVID-19 outbreak, I am temporarily unemployed or not working (e.g. furloughed). (6)

○ I am currently unemployed or out of work, but not directly because of the COVID-19 outbreak. (7)

○ Other (8)

○ Not applicable / rather not say (9)

How many hours per week are you supposed to work according to your employment contract?

_____ hours

On average, how many hours per week do you actually work including any overtime?

_____ hours

What share of your working hours do you currently work from home?

_____ %

What share of your working hours did you use to work from home before the Covid-19 pandemic?

_____ %

When working from home in the past seven days, how often have you experienced situations where you are distracted by the following?

For each item choose one value on the scale from "Never" to "Always". (5-point scale)

Colleagues

Child(ren)

Partner

Housemate(s)

Pet(s)

Social media

Instant messaging

News portals

Noise

Mind wandering

Worries

Household chores

Door bell

[Performance] Below, please indicate how you currently feel about your performance in your job. For each statement choose one value on the scale from "Strongly disagree" to "Strongly agree". (5-point scale)

I adequately complete assigned duties.

I fulfill responsibilities specified in my job description

I perform tasks that are expected of me.

I meet formal performance requirements of the job.

I engage in activities that will directly affect my performance evaluation.

I neglect aspects of my job I am obligated to perform

I fail to perform essential duties.

[Perceived Organizational Support] Listed below are statements that represent some opinions that you may have about the organization you are working at (in case you have several employers, think about the organization you spend most of your working time at). Please indicate to which extend to you agree or disagree with each statement. For each statement choose one value on the scale from "Strongly disagree" to "Strongly agree". (7-point scale)

The organization values my contribution to its well-being.

The organization fails to appreciate any extra effort from me.

The organization would ignore any complaint from me.

The organization really cares about my well-being.

Even if I did the best job possible, the organization would fail to notice.

The organization cares about my general satisfaction at work.

The organization shows very little concern for me.

The organization takes pride in my accomplishments at work.

Are the tasks you do at work rather manual or intellectual?

Choose a value on the scale from 0 ("Rather manual") to 10 ("Rather intellectual").

Are the tasks you perform at work rather routine tasks or creative tasks?

Choose a value on the scale from 0 ("Rather routine") to 10 ("Rather creative").

How much independence do you have in performing your tasks at work?

Choose a value on the scale from 0 ("No independence at all") to 10 ("Complete independence").

Do you supervise other people at work?

- ○ Yes
- ○ No

On average, how often do you communicate with your direct supervisor?

- ○ Daily
- ○ Weekly
- ○ Monthly
- ○ Less than monthly
- ○ Does not apply

On average, how often do you communicate with your colleagues?

- ○ Daily
- ○ Weekly
- ○ Monthly
- ○ Less than monthly
- ○ Does not apply

[Goals] In this part of the questionnaire, we ask you to name at least three work goals that you would like to pursue in the next seven days. Please think of specific tasks that you would like to accomplish.

- ○ Briefly describe your first work goal with at least five words.
  ____
- ○ Briefly describe your second work goal with at least five words.
  ____
- ○ Briefly describe your third work goal with at least five words.
  ____

[Big Five] Please indicate the extent to which each of the following statements reflects **your typical behavior**. For each statement choose one value on the scale from 1 ("Not at all") to 5 ("Very much").

I am the life of the party.

I sympathize with others' feelings.

I get chores done right away.

I have frequent mood swings.

I have a vivid imagination.

I don't talk a lot.

I am not interested in other people's problems.

I often forget to put things back in their proper place.

I am relaxed most of the time

I am not interested in abstract ideas.

I talk to a lot of different people at parties.

I feel other's emotions.

I like order.

I get upset easily.

I have difficulty understanding abstract ideas.

I keep in the background.

I am not really interested in others.

I make a mess of things.

I seldom feel blue.

I do not have a good imagination.

[Self-control] Please indicate the extent to which each of the following statements reflects **your typical behavior**. For each statement choose one value on the scale from 1 ("Not at all") to 5 ("Very much").

I am good at resisting temptation.

I have a hard time breaking bad habits.

I am lazy.

I say inappropriate things.

I do certain things that are bad for me if they are fun.

I refuse things that are bad for me.

I wish I had more self-discipline.

People would say that I have iron self-discipline.

Pleasure and fun sometimes keep me from getting work done.

I have trouble concentrating.

I am able to work effectively toward long-term goals.

Sometimes I can’t stop myself from doing something, even if I know it is wrong.

I often act without thinking through all the alternatives.

[Intrinsic Work Motivation + attention check] Please indicate how much each of the following statements reflects **how you typically are**. For each statement choose one value on the scale from 1 ("Not at all") to 5 ("Very much").

I work because I derive much pleasure from learning new things.

I work for the satisfaction I experience from taking on interesting challenges.

I work for the satisfaction I experience when I am successful at doing difficult tasks.

Please select the middle of the scale here. This statement checks your attention.

How satisfied are you with your life, all things considered?

Please choose one value on the scale from 0 ("Extremely dissatisfied") to 10 ("Extremely satisfied").

For each domain choose one value on the scale from 0 ("Extremely dissatisfied") to 10 ("Extremely satisfied"). **How satisfied are you with…**

...your health?

...your sleep?

...your job?

...your income?

...your free time?

...working from home?

...your household chores?

[Depression] Below you find a list of the ways you might have felt or behaved during the past week. On a scale from "Never" to "Always", please indicate **how much of the time during the past seven days**...

…you felt depressed?

…you felt that everything you did was an effort?

…your sleep was restless?

…you were happy?

…you felt lonely?

…you enjoyed life?

…you felt sad?

…you could not get going? (in the sense of ‘felt lethargic and lacked motivation’)

What sex were you assigned at birth, such as on an original birth certificate?

○ Male

○ Female

○ Prefer not to say

How old are you?

______ years

What is the highest degree or level of education you have completed?

○ Some high school, no diploma

○ High school graduate

○ Some college, no degree

○ Associate degree

○ Bachelor's degree

○ Master's degree

○ Professional degree

○ Doctorate degree

What is your current income? Please indicate the answer that includes your entire personal income (previous year) before taxes.

○ Less than £10,000

○ £10,000 - £19,999

- ○ £20,000 - £29,999
- ○ £30,000 - £39,999
- ○ £40,000 - £49,999
- ○ £50,000 - £59,999
- ○ £60,000 - £69,999
- ○ £70,000 - £79,999
- ○ £80,000 - £89,999
- ○ £90,000 - £99,999
- ○ £100,000 - £149,999
- ○ More than £150,000
- ○ Rather not say

How many of your household members are below the age of 14? (0-5, more than 5)

Experimental instructions

The survey part of this study is now over. In the next section, which will only take a few minutes, we describe one behavioral strategy. We would like to ask you to follow this strategy for the next seven days. **Please read these instructions very carefully.**

(Self-control strategies) Below are the instructions for a behavioral strategy. Please try to follow them for one week starting tomorrow as much as you can. We will contact you in a week and ask you to fill in a survey about your experiences, behaviors and feelings this week.

- (Treatment Situation modification) For the next week, every workday before you start working, remove any distractions from your workspace. For example, close chat programs, log out of distracting social media websites, switch off your phone, or ask your household members not to disturb you during your work time. For the following week, please try to follow this strategy every workday as much as you can.

- (Treatment Goal reminder) For the next week, when you face distractions while working, remind yourself of your work goals. For example, think about the goals that you have set for yourself previously in this questionnaire. For the following week, please try to follow this strategy every workday as much as you can.

- (Treatment Give in later) For the next week, make yourself a promise to give into distractions later, after you are done with your work task. For example, promise yourself to check your phone for private messages or your social media account when you are done with the task that you are working on.

Now take some time to think about the typical factors that distract you from work. Please use the space below to specify how you will modify your workplace to prevent these factors from distracting you during the following week.

____

____

____

(Control group) For the next week, try to keep up the usual work schedule and routines the way you were following the past months.

For the following week, please try to follow this strategy every workday as much as you can.

Please use the space below to write down what a typical work day looks like for you. For example, you could write what time you start work, how often you take breaks, how much time you spend in meetings, when you stop work for the day.

Please confirm the statement below.

○ **I confirm that I will follow the strategy as described above starting from tomorrow and every workday for the period of 7 days.**

Wave 2

How many hours did you work for your paid job the past seven days?

_____ hours

What share of your working hours did you work from home in the past seven days?

Please indicate a number between 0 and 100%.

_____ %

Did you have any days off from work during the last seven days (not counting the weekend)?

○ No

○ Yes

How many days did you have off from work during the last seven days (not counting the weekend)?

○ 1 day

○ 2 days

○ 3 days

○ 4 days

○ 5 days

[Performance] Below, please indicate how you currently feel about your performance in your job. For each statement choose one value on the scale from "Strongly disagree" to "Strongly agree". (5-point scale)

I adequately complete assigned duties.

I fulfill responsibilities specified in my job description

I perform tasks that are expected of me.

I meet formal performance requirements of the job.

I engage in activities that will directly affect my performance evaluation.

I neglect aspects of my job I am obligated to perform

I fail to perform essential duties.

How satisfied are you with your life, all things considered?

Please choose one value on the scale from 0 ("Extremely dissatisfied") to 10 ("Extremely satisfied").

For each domain choose one value on the scale from 0 ("Extremely dissatisfied") to 10 ("Extremely satisfied"). **How satisfied are you with…**

...your health?

...your sleep?

...your job?

...your income?

...your free time?

...working from home?

...your household chores?

[Depression] Below you find a list of the ways you might have felt or behaved during the past week. On a scale from "Never" to "Always", please indicate **how much of the time during the past seven days**...

…you felt depressed?

…you felt that everything you did was an effort?

…your sleep was restless?

…you were happy?

…you felt lonely?

…you enjoyed life?

…you felt sad?

…you could not get going? (in the sense of ‘felt lethargic and lacked motivation’)

In the first part of the study we asked you to describe three work goals that you wanted to pursue during the coming week. **Think about your goal progress in the last seven days and answer the questions below.**

Your work goal 1/2/3 was: […]

On a scale from „Strongly disagree“ to „Strongly agree" indicate how much you agree or disagree with the following statements about your work goal 1/2/3.

I have made a great deal of progress concerning this goal.

I have hardly made any progress in the attempt of advancing in this goal.

I have had quite a lot of success in pursuing this goal.

Many of my efforts in carrying out this goal have failed.

Many things happened that have obstructed this goal.

It is much harder than I thought to materialize this goal.

Think of your working from home during the past seven days.

[Strategy use + attention check] How often did you follow the behaviors described below when you felt distracted?

Please indicate for each behavior one value on the scale from "Never" to "Always". (5-point scale)

I removed any distractions from my workspace before starting to work.

I reminded myself of my work goals when I got distracted.

I made myself a promise to give into distraction later after I was done with my work.

I reminded myself of why it was bad for me to let myself get distracted.

I simply resisted the distraction.

I gave in to the distraction.

Please select the middle of the scale here. This statement checks your attention.

It is important for us to ensure that our study instructions are as clear as possible.

Please think back to Part 1 of this study where we asked you to follow a behavioral strategy. (We have also sent you a reminder about it).

What was your assigned strategy in Part 1?

Please select one.

- ○ To remove any distractions from my workspace before starting to work.
- ○ To remind myself of my work goals when I get distracted.
- ○ To make myself a promise to give into distraction later after I am done with my work.
- ○ To remind myself of why it was bad for me to let myself get distracted.
- ○ To simply resist distractions.
- ○ To give into distractions at work.

○ To follow my usual routines at work.

○ I don't remember the strategy.

In Part 1 we asked you to follow this strategy: […]

For the next week, every workday before you start working, remove any distractions from your workspace. For example, close chat programs, log out of distracting social media websites, switch off your phone, or ask your household members not to disturb you during your work time.

How difficult did you find it to follow this strategy?

Please choose one value on the scale from "Extremely easy" to "Extremely difficult".

○ Extremely easy

○ Somewhat easy

○ Neither easy nor difficult

○ Somewhat difficult

○ Extremely difficult

To what extent have you been using this strategy during the past week? Please choose one value on the scale from "Never" to "Always".

○ Never

○ Sometimes

○ About half the time

○ Most of the time

○ Always

Did you find the strategy useful? Please choose one value on the scale from "Not at all useful" to "Extremely useful".

○ Not at all useful

○ Slightly useful

○ Moderately useful

○ Very useful

○ Extremely useful

Wave 3

How many hours per week are you supposed to work according to your employment contract?

_____ hours

On average, how many hours per week do you actually work including any overtime?

_____ hours

What share of your working hours do you currently work from home?

_____ %

What share of your working hours did you use to work from home before the Covid-19 pandemic?

_____ %

On how many days do you usually work for your paid job each week? (1-7 days)

When working from home in the past seven days, how often have you experienced situations where you were distracted by the following?

For each item choose one value on the scale from "Never" to "Always". (5-point scale)

When working from home in the past seven days, how often have you experienced situations where you were distracted by the following?

For each item choose one value on the scale from "Never" to "Always".

Colleagues

Child(ren)

Partner

Housemate(s)

Pet(s)

Social media

Instant messaging

News portals

Noise

Mind wandering

Worries

Household chores

Door bell

[Performance] Below, please indicate how you currently feel about your performance in your job. For each statement choose one value on the scale from "Strongly disagree" to "Strongly agree". (5-point scale)

I adequately complete assigned duties.

I fulfill responsibilities specified in my job description

I perform tasks that are expected of me.

I meet formal performance requirements of the job.

I engage in activities that will directly affect my performance evaluation.

I neglect aspects of my job I am obligated to perform

I fail to perform essential duties.

[Perceived Organizational Support] Listed below are statements that represent some opinions that you may have about the organization you are working at (in case you have several employers, think about the organization you spend most of your working time at). Please indicate to which extend to you agree or disagree with each statement. For each statement choose one value on the scale from "Strongly disagree" to "Strongly agree". (7-point scale)

The organization values my contribution to its well-being.

The organization fails to appreciate any extra effort from me.

The organization would ignore any complaint from me.

The organization really cares about my well-being.

Even if I did the best job possible, the organization would fail to notice.

The organization cares about my general satisfaction at work.

The organization shows very little concern for me.

The organization takes pride in my accomplishments at work.

[Strategy use] How often did you follow the behaviors described below when you felt distracted?

Please indicate for each behavior one value on the scale from "Never" to "Always". (5-point scale)

I removed any distractions from my workspace before starting to work.

I reminded myself of my work goals when I got distracted.

I made myself a promise to give into distraction later after I was done with my work.

I reminded myself of why it was bad for me to let myself get distracted.

I simply resisted the distraction.

I gave in to the distraction.

Are the tasks you do at work rather manual or intellectual?

Choose a value on the scale from 0 ("Rather manual") to 10 ("Rather intellectual").

Are the tasks you perform at work rather routine tasks or creative tasks?

Choose a value on the scale from 0 ("Rather routine") to 10 ("Rather creative").

How much independence do you have in performing your tasks at work?

Choose a value on the scale from 0 ("No independence at all") to 10 ("Complete independence").

Do you supervise other people at work?

○ Yes

○ No

On average, how often do you communicate with your direct supervisor?

- ○ Daily
- ○ Weekly
- ○ Monthly
- ○ Less than monthly
- ○ Does not apply

On average, how often do you communicate with your colleagues?

- ○ Daily
- ○ Weekly
- ○ Monthly
- ○ Less than monthly
- ○ Does not apply

[Intrinsic Work Motivation + attention check] Please indicate how much each of the following statements reflects **how you typically are**. For each statement choose one value on the scale from 1 ("Not at all") to 5 ("Very much").

I work because I derive much pleasure from learning new things.

I work for the satisfaction I experience from taking on interesting challenges.

I work for the satisfaction I experience when I am successful at doing difficult tasks.

Please select the middle of the scale here. This statement checks your attention.

How satisfied are you with your life, all things considered?

Please choose one value on the scale from 0 ("Extremely dissatisfied") to 10 ("Extremely satisfied").

For each domain choose one value on the scale from 0 ("Extremely dissatisfied") to 10 ("Extremely satisfied"). **How satisfied are you with…**

...your health?

...your sleep?

...your job?

...your income?

...your free time?

...working from home?

...your household chores?

How satisfied are you currently with your work performance working from your organization's premises? (5-point scale)

[Depression] Below you find a list of the ways you might have felt or behaved during the past week. On a scale from "Never" to "Always", please indicate **how much of the time during the past seven days**...

…you felt depressed?

…you felt that everything you did was an effort?

…your sleep was restless?

…you were happy?

…you felt lonely?

…you enjoyed life?

…you felt sad?

…you could not get going? (in the sense of 'felt lethargic and lacked motivation')

How many of your household members are below the age of 14? (0-5, more than 5)

During the COVID-19 pandemic, are you working from home?

- ○ Yes, I am working from home every day. I rarely worked from home before COVID-19 (less than 1 day a week).
- ○ Yes, I am working from home every day. I sometimes worked from home before COVID-19 (1 day a week or more).
- ○ Yes, I am working from home every day. I always worked from home before COVID-19.

○ Yes, I am sometimes working from home, but still commuting to my workplace on other days.

○ No, I am still commuting to work every day, even during the COVID-19 pandemic.

○ Due to the COVID-19 outbreak, I am temporarily unemployed or not working (e.g. furloughed).

○ I am currently unemployed or out of work, but not directly because of the COVID-19 outbreak.

○ Other

○ Not applicable / rather not say

References

Eisenberger, R., Huntington, R., Hutchison, S., and Sowa, D. (1986). Perceived organizational support. Journal of Applied Psychology, 71(3):500–507.

Milyavskaya, M., Saunders, B., and Inzlicht, M. (2020). Self-control in daily life: Prevalence and effectiveness of diverse self-control strategies. Journal of Personality.

Tremblay, M. A., Blanchard, C. M., Taylor, S., Pelletier, L. G., and Villeneuve, M. (2009). Work Extrinsic and Intrinsic Motivation Scale: Its value for organizational psychology research. Canadian Journal of Behavioural Science/Revue canadienne des sciences du comportement, 41(4):213–226.
